# The prevalence of phenylketonuria (PKU) and hyperphenylalaninemia (HPA) in Iran: a systematic review and meta-analysis

**DOI:** 10.1186/s13023-026-04255-z

**Published:** 2026-02-25

**Authors:** Samira Faraji, Seyed Mohammad Tabatabaee Jabali, Mohammad Hasan Abolhasani, Davoud Amirkashani, AliAkbar Haghdoost, Mahdi Shadnoush, Saeedeh Talebi, Golnaz Ranjbar, Saeid Talebi, Maryam Mahmoudi, Peyman Eshraghi, Tayebeh Najafimoghadam, Bahareh Lesani Gouya, Mohsen Nematy, Abdolreza Norouzy

**Affiliations:** 1 Department of Nutrition, Faculty of Medicine, Mashhad University of Medical Sciences, Mashhad, Iran; 2 Department of Sport and Exercise Medicin, School of Medicine, Iran University of Medical Sciences, Tehran, Iran; 3 Minimally Invasive Surgery Research & Training Center of Excellence Iran, University of Medical Sciences, Tehran, Iran; 4 Department of Clinical Nutrition, School of Medicine, Iran University of Medical Sciences, Tehran, Iran; 5 Department of Pediatric Endocrinology, Aliasghar Children’s Hospital, Aliasghar Clinical Research, Tehran, Iran; 6 Development Center, Iran University of Medical Sciences, Tehran, Iran; 7 Modeling in Health Research Center, Institute for Futures Studies in Health, Kerman University of Medical Sciences, Kerman, Iran; 8 Department of Clinical Nutrition & Dietetics, Faculty of Nutrition Science and Food Technology, National Nutrition and Food Technology Research Institute, Shahid Beheshti University of Medical Sciences, Tehran, Iran; 9 Department of Biological and Environmental Sciences, Faculty of Life and Medical Sciences, University of Hertfordshire, Hatfield, UK; 10 Department of Medical Genetics and Molecular Biology, Faculty of Medicine, Iran University of Medical Sciences, Tehran, Iran; 11 Department of Cellular and Molecular Nutrition, School of Nutritional Sciences and Dietetics, Tehran University of Medical Sciences, Tehran, Iran; 12 Pediatric Endocrinology Department, Akbar Hospital, Faculty of Medicine, Mashhad University of Medical Sciences, Mashhad, Iran; 13 Iran University of Medical Science, Tehran, Iran; 14 Department of Health, Iran University of Medical Sciences, Tehran, Iran; 15 Metabolic Syndrome Research Center, Mashhad University of Medical Sciences, Mashhad, Iran

**Keywords:** Phenylketonurias, PKU, Phenylketonuria, Hyperphenylalaninemia, HPA, Iran, Prevalence, Incidence, Meta-analysis

## Abstract

**Background:**

Phenylketonuria (PKU) is one of the common Inborn Errors of Metabolism diseases, that is caused by a phenylalanine hydroxylase (PAH) deficiency or cofactor tetrahydrobiopterin. This systematic review and meta-analysis aimed to investigate the prevalence of PKU in Iran.

**Methods:**

The protocol was registered in PROSPERO (CRD42024540811). The MOOSE protocol and the PRISMA guidelines were used. The Web of Sciences, PubMed/ Medline, Sciences Direct, Google Scholar, Scopus, Civilica, IranDoc, and SID databases were searched on 31/03/2024. The I^2^ index and Q test were used to check heterogeneity. Comprehensive meta-analysis software (CMA ver. 2) was used (*P* < 0.05 is considered significant). The prevalences were reported in 100,000 neonates at national and provincial levels.

**Results:**

Finally, 20 studies with 1,992,090 Iranian neonates were included. The prevalence of screen-positive cases was 75.6 (95% CI: 48.1–118.72). The prevalence of confirmed PKU was 16.7 (95% CI: 13.6– 20.5); this prevalence in girls and boys was 15.2 (95% CI: 5.2–44.2) and 9.8 (95% CI: 3.2– 29.8), respectively. 53% of the cases had Hyperphenylalaninemia (HPA). The prevalence of HPA and classical PKU was estimated at 8.9 (95% CI: 5.9–13.41) and 8.0 (95% CI: 5.1–12.59), respectively. Subgroup analysis was performed based on region, province, and study quality to discover the source of heterogeneity. In addition, mixed effects meta-regression was used to find the relationship between continuous variables. Sensitivity analysis showed that the pooled estimate was robust.

**Conclusions:**

It seems that the screening program in Iran was effective and detected almost all PKU cases in the first few days of their lives. This information showed that the PKU prevalence is relatively higher than in most parts of the world, thus their prevalence should be controlled.

**Supplementary Information:**

The online version contains supplementary material available at 10.1186/s13023-026-04255-z.

## Introduction

Phenylketonuria (PKU; OMIM 261600, also known as Phenylalanine hydroxylase (PAH; E.C.1.14.16.1) deficiency) is an Inborn Errors of Metabolism (IEM) resulting in some disorders in biochemical metabolic and a series of clinical symptoms in newborn babies [1]. Phenylalanine hydroxylase (PAH) gene mutations cause this autosomal recessive disorder, which affects the body’s levels of phenylalanine (Phe), an essential amino acid [2, 3]. PAH is a liver enzyme that requires the cofactor tetrahydrobiopterin (BH4) to convert Phe to tyrosine. Deficiency of PAH or its cofactor BH4 leads to the accumulation of excess phenylalanine, whose toxic effects can cause severe and irreversible mental disability if left untreated [4]. BH4 deficiency is the reason for Phenylketonuria and Hyperphenylalaninemia in about 1–2% of patients with PKU, in which the pharmaceutical form of tetrahydrobiopterin, i.e., Sapropterin Dihydrochloride (Kuvan) is prescribed in the form of tablets or powder at a dose of 20 − 5 mg/kg per day [5]. A BH4 loading test should be conducted to monitor BH4 responsiveness, and the 20–29% decrease in blood Phe is a partial response to BH4. A reduction of more than 30% is desirable in response to BH4 [6].

The prevalence of PKU varies greatly among ethnicities and geographic regions. Around the world, PKU has an estimated incidence of 1 in 23,930 live births and affects about 0.45 million people, with at least two-thirds requiring treatment [7]. The incidence is generally higher in white or East Asian populations in terms of ethnicity (1 in 10,000 to 15,000 live births). Geographically, the incidence of PKU is lowest in Asian countries (except China) and highest in European and Middle Eastern countries. In the Middle East, the highest incidence is in Egypt, Iran, and Jordan, with approximately 1 in 5,000 live births [8, 9]. According to a recent meta-analysis study conducted from 1964 to 2017, the highest prevalence (38.13) was reported in Turkey and the lowest (0.3) in Thailand. In addition, the overall incidence of this disease worldwide was reported to be 6.002 per 100,000 live births [10].

According to a meta-analysis and systematic review study, the prevalence of PKU in Iran was reported to be 16.5 per 100,000 live births up to 2019 [11]. The incidence of phenylketonuria in southeast, west, north-eastern, northern of Iran was calculated 1.27 per 10,000 (2012–2019), 1.91 per 10,000 (2006–2016), 57 per 1,000,000 (2012–2013), 0.66 in 10,000 live births (2007–2015; Hyperphenylalaninemia (HPA): 1 in 33,937, mild PKU: 1 in 45,249, and severe PKU: 1in 67,874) [12–15].

Many countries conduct routine newborn screening (NBS) programs to detect elevated Phe concentrations due to the severe consequences of untreated phenylalanine hydroxylase deficiency [16, 17]. NBS has allowed early diagnosis and successful treatment with the phenylalanine-restricted diet. The first NBS program appeared in the United States in the early 1960s [18]. PKU screening program was started in Iran in 2015 [19]. This timely diagnosis significantly reduces the financial and family burden across the country and leads to early intervention to prevent further complications from PKU [20, 21]. According to existing guidelines, the Guthrie test should be performed three to five days after birth, and 4 mg/dl or higher High-performance liquid chromatography (HPLC method) confirming test should be performed for neonates with phenylalanine levels [11].

The PKU classification scheme has recently been defined as no treatment required but BH4, diet, or both [22]. The primary treatment, nutritional therapy, should be conducted under the supervision of a team of pediatricians, nutritionists, psychologists, nurses, and social workers, which will be effective when it is started before the third week of the baby’s life [23]. Dietary therapy for PKU includes a Phe-restricted diet and a medical food based on Phe-free amino acids. Medical foods for PKU provide patients with all other essential amino acids, tyrosine, fat, carbohydrates, and micronutrients [24]. The primary considerations in regulating the diet of these patients are providing sufficient amounts of energy, protein, and other nutrients, limiting Phe, and using tyrosine supplements based on age, disease genotype, growth rate, and energy required to maintain blood Phe concentration in the range of 2-6 mg/dl, which is applied through the use of ready-made formula foods and the consumption of low-protein and low-protein foods so that it is possible to achieve optimal growth and brain development for the child [23]. As a result, classic and mild PKU require dietary protein restriction and supplementation with Phe-free amino acid mixtures to maintain blood Phe levels within a safe range. In contrast, no dietary limitation is necessary in non-PKU HPA [25].

Despite several studies in different cities of Iran regarding the prevalence of PKU using screening programs, no study has systematically investigated the prevalence of PKU in Iran. Therefore, this systematic review and meta-analysis were conducted to examine the prevalence of PKU in Iran. Meta-analysis is a statistical analysis that combines the results of several scientific studies. Pooled data from meta-analyses are usually more helpful than results from narrative reviews. The decisions are transparent in a meta-analysis, and the statistical analysis objectively measures the integrated quantitative evidence. It is an essential method for synthesizing studies and provides an accurate and universal answer according to all current knowledge. Also considering that NBS is a successful public health system used for the early detection of hazardous hereditary diseases, including PKU, and failure to diagnose these diseases in time can lead to mental retardation, permanent disability, and even death. Therefore, awareness of the prevalence of this hereditary disease and the extent of children’s involvement in this manageable and treatable disease can increase attention to this vulnerable group of society as well as improve its prevention and control.

## Methods

### Study protocol

The study protocol of this systematic review and meta-analysis was registered in PROSPERO (International Prospective Register of Systematic Reviews) before it was performed (CRD42024540811). Available from: https://www.crd.york.ac.uk/prospero/display_record.php?ID=CRD42024540811. The MOOSE (Meta-analyses Of Observational Studies in Epidemiology) protocol and the PRISMA (Preferred Reporting Items for Systematic Reviews and Meta-analysis) guidelines were used to design and report the study [26, 27]. Considering the type of study, which was a meta-analysis, there was no need for the ethics committee’s approval.

### Search strategy

The Sciences Direct, PubMed/Medline, Web of Sciences, Google Scholar, Scopus, Civilica (https://www.civilica.com/), Iranian Research Institute for Information Science and Technology (IranDoc( (https://irandoc.ac.ir), and Scientific Information Database (SID) (http://www.sid.ir/), databases were searched on 31/03/2024 for relevant literature. The search was conducted without time and language restrictions. A comprehensive search in the mentioned international online databases was performed by S. Faraji and A.B. Norouzy qualified researchers and experts in the field of database searching. The search strategy in databases was defined as follows based on the purpose of the present meta-analysis:

1. (((Mortality[Title]) OR (Prevalence[Title])) OR (Incidence[Title])) AND (((((((PKU[Title]) OR (Phenylketonuria[Title])) OR (Phenylketonurias [MeSH Terms])) OR (Hyperphenylalaninemia[Title])) OR (BH4 Deficiency[Title])) OR (Tetrahydrobiopterin Deficiency[Title])) OR (DHPR Deficiency[Title])) OR (Dihydropteridine Reductase Deficiency[Title]))
2. (((((((PKU[Title]) OR (Phenylketonuria[Title])) OR (Phenylketonurias [MeSH Terms])) OR (Hyperphenylalaninemia[Title])) OR (BH4 Deficiency[Title])) OR (Tetrahydrobiopterin Deficiency[Title])) OR (DHPR Deficiency[Title])) OR (Dihydropteridine Reductase Deficiency[Title])) AND (Iran[Title])

The search keywords were adjusted based on the detailed specifications and differences in the syntactic rules of each database to search the Iranian database to find Persian articles. The reference lists of all retrieved articles were manually reviewed to identify all potential studies. In addition, a reference check was performed, as well as two articles [28, 29] related to the topic of the article were entered. Corresponding authors were contacted to find the full text of some articles. We sent an email to the corresponding authors.

### Inclusion criteria (PICO)

Inclusion criteria were PICO (Patient, Problem, or Population; Intervention, Prognostic Factor, and Exposure; Comparison, Control, or Intervention; Outcome): (1) *P*opulation: all Iranian children in both genders; (2) *I*ntervention: diagnosis of PKU-based NBS program by HPLC method, Colorimetric method, Fluorometric method, Enzyme-linked immunosorbent assay (ELISA method); (3) *C*omparison: variable aimed for prevalence and incidence of PKU and HPA such as region, province, year of study, gender, etc.; (4) *O*utcome: Prevalence and incidence of PKU and HPA.

### Exclusion criteria

Exclusion criteria included: (1) Reviews, comments, animal studies, letters to the editor without quantitative data, case studies, randomized control trials and case-control studies, and meta-analysis; (2) being irrelevant; (3) duplicate studies; (4) non-Iranian studies; (5) The sample size related to Iranian people in combination with non-Iranians (such as Afghanistanis); (6) Studies on specific patients (such as mentally retarded patients); (7) Non-random samples; (8) Articles in languages ​​other than English and Persian; (9) Failure to mention the number of patients in each group (Screen-positive cases, Confirmed PKU, HPA, and Classical PKU or Severe PKU; 10) Not mentioning the sample size.

### Data collection

The title and text of the reviewed articles and the studies conducted outside of Iran were excluded from the study. Peer-reviewed epidemiological studies that directly reported the prevalence and incidence of PKU based on screening newborn populations were included. All articles were screened in terms of title and abstract. Then, the full text of the articles was evaluated according to the inclusion and exclusion criteria. Finally, the discussion and review of the differences raised were conducted in the presence of all the authors.

### Operational definitions

Based on various definitions and guidelines, phenylketonuria (PKU) is divided into different types: Classical PKU (Phe level above 20 mg/dl), Moderate PKU (Phe level 15-20 mg/dl), Mild PKU (Phe level 10-15 mg/dl), and Hyperphenylalaninemia (HPA) (Phe level 2 or 4-10 mg/dl). However, given that meta-analysis and systematic review studies extract and combine data to produce a summary result, and according to the data from the studies included in this article, PKU division was not performed for data below 20 mg/dl and there was no data in the number of studies [30].

Even, there was a different classification and definition of phenylalanine levels in the included studies such as: Phe level <4 mg/dl, Phe level 4-19.9 mg/dl, and Phe level ≥ 20 mg/dl [31] or Mild PKU = Phe level 6-20 mg/dl and Classical PKU = Phe-level > 20 mg/dl [32] or HPA = Phe level ≥ 2-10 mg/dl, Mild PKU = Phe level 10-20 mg/dl and Severe PKU = Phe level ≥ 20 mg/dl [13] or Mild HPA= Serum Phe 2-20 mg/dl and Classical PKU = Phe level > 20 mg/dl [33, 34].

We had to divide the data into two subgroups to consider and collect all the results of the studies: Phe level above 20 mg/dl and Phe level below 20 mg/dl. Therefore, based on consultation with a Pediatric Endocrinologist, we had to choose a name and definition for it.

In this study phenylalanine levels were operationally categorized into two groups: (1) HPA: Phe level 2 or 4-20 mg/dl and (2) Classical PKU or Severe PKU: Phe level above 20 mg/dl. Screen-positive cases or referral cases suspected of PKU were defined as subjects with Phe levels above 2 or 4 mg/dl on the NBS program. Screen-positive cases were diagnosed as confirmed PKU cases after confirmatory tests.

This classification was adopted to facilitate statistical analysis and was based on threshold values referenced in prior studies that used 20 mg/dl as a clinical marker of disease severity. It should be noted that this operational definition does not replace internationally accepted classifications, but rather serves as a framework specific to the analytical aims of the present research.

### Data extraction and management

Data extracted by the authors included the first author’s name, year of publication, study location, period of data collection, sample size (total, boys and girls), number of cases (total, boys and girls), type of study, age at sampling, prevalence of each type of PKU (Screen-positive cases, Confirmed PKU, Classic PKU or Severe PKU, and HPA), consanguinity percent, screening percent and timely screening percent (within 3 to 5 days of birth). After extracting the data from the articles (XP professional edition; Microsoft, Redmond, Washington, USA), these data were imported into the Excel software. Duplicate studies were removed through EndNote™ Software, and the full text of the article was referred to for ambiguous cases or contacted by the corresponding author or the first author. All stages of the study were performed independently by two authors. Any disagreement between the two investigators was resolved in consultation with a third reviewer or clinical consultant. All results were entered twice in the data extraction form to avoid data entry errors. Additionally, percentages were used to report PKU prevalence and new disease cases per 100,000 live births to report PKU incidence to ensure accurate comparisons between results.

### Assessment of methodological quality

The quality of the studies was evaluated in terms of implementation and reporting methods. For cross-sectional/prevalence studies, the quality of the articles was classified into three categories: low (0–5 score), medium (6–7 score), and high (8–9 score) based on the modified Newcastle-Ottawa Scale (NOS) [35]. We had no studies with poor quality to exclude it.

### Statistical analysis

The heterogeneity of the studies was determined by Cochran’s Q and I^2^ tests (*P* < 0.10 is considered significant). Based on the I^2^ test, the I^2^ index less than 25%, 25–49%, 50–75%, and more than 75% is defined as low, moderate, considerable, and high heterogeneity, respectively. The fixed effects model is used in cases of low heterogeneity, and the random effects model is used in higher heterogeneities. Therefore, the random effects model was used to estimate the prevalence of PKU in this meta-analysis. Subgroup analysis was performed based on region, province, year, and study quality to discover the source of heterogeneity. Sensitivity analysis was used to measure the power of the overall estimate at one time. The girls-boys odds ratio (OR) was used to show the effect of gender on PKU. The OR and 95% CI of girls-boys show the impact of gender on PKU. In this meta-analysis, sensitivity analysis was also performed to confirm the stability of the data. Begg and Egger tests were used to evaluate the publication bias. The data were analyzed by Comprehensive meta-analysis (CMA ver. 2) software (*P* < 0.05 is considered significant). The mixed effects meta-regression was used to find the relationship between continuous variables.

For each study included in the meta-analysis, data regarding the number of patients with PKU and the total population were extracted. These data are typically found in the Results, Tables, or Descriptive Statistics sections.

So, for each study, we needed two main pieces of data:

3. The number of people diagnosed with PKU.
4. The total population or number of individuals in the study group (Sample size of each study).

Then, for each study, we calculated the prevalence of the disease in 100,000 by the following formula:

Prevalence (Per 100,000) = (Number of PKU cases/ Total population) ×100,000.

Similarly, we also calculated the prevalence of PKU for each region. After calculating the prevalence of PKU per 100,000 people (to enable data comparability), the data were analyzed by Comprehensive meta-analysis (CMA ver. 2) software (*P* < 0.05 was considered significant).

## Results

### Search results and characteristics of studies

**Fig. 1:**
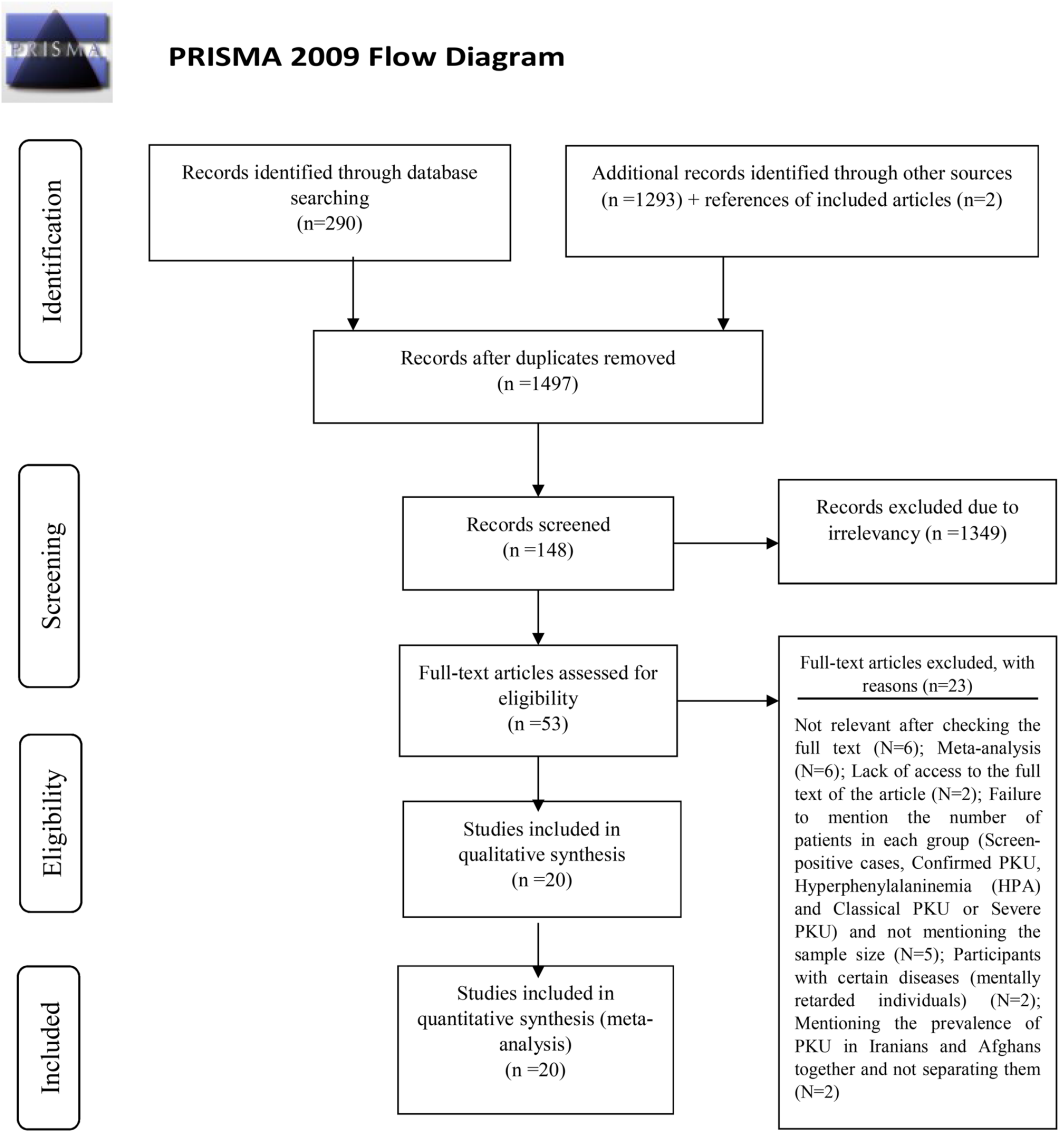
PRISMA flowchart. From: Moher D, Liberati A, Tetzlaff J, Altman DG, The PRISMA Group (2009). Preferred Reporting Items for Systematic Reviews and Meta-Analyses: The PRISMA Statement. PLoS Med 6(6): e1000097. doi:10.1371/journal.pmed1000097 For more information, visit http://www.consort-statement.org/

The study selection flow diagram was based on the PRISMA 2009 flow diagram (Fig. 1). After a systematic search in databases (Sciences Direct, PubMed/Medline, Web of Sciences, and Scopus), 290 related articles were identified. Searching the databases Google Scholar, Civilica, IranDoc, and SID for articles and abstracts published in Persian, 1293 articles were found. In addition, two related articles by Golbahar J. et al. [28] and Saadatpour Y. et al. [29] were found through reference checks. A total of 1585 articles were included in the study, and 22 articles were included based on the inclusion/exclusion criteria after removing duplicate articles through the EndnoteTM software and checking the title, abstract, and, finally, the full text of the article. The systematic review included 20 articles, two excluded due to lack of access to the full text. Studies by Motamedi, N. et al. [14], Rezabighi Davarani, F. et al. [36], Rezabighi Davarani, F. et al. [12], Abbaskhanian, A. et al. [13] Saadatpour, Y. et al. [29] Saadatinasab, Z. et al. [37], and Ganji, F. et al. [38] were considered as more than one study. Because they were investigated in different populations and different years. These studies estimated prevalence for over a year in other regions. Therefore, each study was considered separately to determine the slope of prevalence in different years and the region with the highest or lowest prevalence [39]. Quantitative data obtained from eligible studies are summarized in Table 1.

**Table 1:** Description of studies included in the meta-analysis

Reference*	First author, Year of publication	Study location	Type of Study	Age at sampling	Period of Data collection	Consanguinity%	Sample size			Number of cases			Incidence	Prevalence	Screen-positive cases	Confirmed PKU^a^	Classic PKU or Severe PKU	HPA (Hyperphenylalaninemia)	Screening %	Timely screening % (within 3 to 5 days of birth)	Quality	Remarks
							All	Boy	Girl	All	Boy	Girl										
[14]	Motamedi, N. 2017	Lorestan	Observational-descriptive study	3–5 Days	2006	-	33,284	-	-	-	-	-	1.17 in 10,000	-	-	4	-	-	-	-	High	-
[14]	Motamedi, N. 2017	Lorestan	Observational-descriptive study	3–5 Days	2007	-	33,890	-	-	-	-	-	1.66 in 10,000	-	-	5	-	-	-	-	High	-
[14]	Motamedi, N. 2017	Lorestan	Observational-descriptive study	3–5 Days	2008	-	34,045	-	-	-	-	-	2.05 in 10,000	-	-	7	-	-	-	-	High	-
[14]	Motamedi, N. 2017	Lorestan	Observational-descriptive study	3–5 Days	2009	-	35,969	-	-	-	-	-	1.47 in 10,000	-	-	5	-	-	-	-	High	-
[14]	Motamedi, N. 2017	Lorestan	Observational-descriptive study	3–5 Days	2010	-	35,011	-	-	-	-	-	1.82 in 10,000	-	-	6	-	-	-	-	High	-
[14]	Motamedi, N. 2017	Lorestan	Observational-descriptive study	3–5 Days	2011	-	35,799	-	-	-	-	-	2.87 in 10,000	-	-	10	-	-	-	-	High	-
[14]	Motamedi, N. 2017	Lorestan	Observational-descriptive study	3–5 Days	2012	-	37,154	-	-	-	-	-	1.62 in 10,000	-	-	6	-	-	-	-	High	-
[14]	Motamedi, N. 2017	Lorestan	Observational-descriptive study	3–5 Days	2013	-	37,944	-	-	-	-	-	1.07 in 10,000	-	-	4	-	-	-	-	High	-
[14]	Motamedi, N. 2017	Lorestan	Observational-descriptive study	3–5 Days	2014	-	39,388	-	-	-	-	-	3.86 in 10,000	-	-	15	-	-	-	-	High	-
[14]	Motamedi, N. 2017	Lorestan	Observational-descriptive study	3–5 Days	2015	-	38,585	-	-	-	-	-	1.37 in 10,000	-	-	5	-	-	-	-	High	-
[14]	Motamedi, N. 2017	Lorestan	Observational-descriptive study	3–5 Days	2016	-	36,585	-	-	-	-	-	2.02 in 10,000	-	-	7	-	-	-	-	High	-
[36]	Rezabeigi, D. 2023	Sirjan	Cross-sectional retrospective study	3–5 Days	2012	-	5,533	-	-	-	-	-	0 in 10,000	-	7	0	0	0	-	-	High	-
[36]	Rezabeigi, D. 2023	Sirjan	Cross-sectional retrospective study	3–5 Days	2013	-	5,852	-	-	-	-	-	5.12 in 10,000	-	8	3	3	0	-	-	High	-
[36]	Rezabeigi, D. 2023	Sirjan	Cross-sectional retrospective study	3–5 Days	2014	-	5,971	-	-	-	-	-	0 in 10,000	-	2	0	0	0	-	-	High	-
[36]	Rezabeigi, D. 2023	Sirjan	Cross-sectional retrospective study	3–5 Days	2015	-	6,147	-	-	-	-	-	1.62 in 10,000	-	3	1	1	0	-	-	High	-
[36]	Rezabeigi, D. 2023	Sirjan	Cross-sectional retrospective study	3–5 Days	2016	-	5,936	-	-	-	-	-	0 in 10,000	-	4	1	0	1	-	-	High	-
[36]	Rezabeigi, D. 2023	Sirjan	Cross-sectional retrospective study	3–5 Days	2017	-	5,316	-	-	-	-	-	0 in 10,000	-	2	0	0	0	-	-	High	-
[36]	Rezabeigi, D. 2023	Sirjan	Cross-sectional retrospective study	3–5 Days	2018	-	5,220	-	-	-	-	-	1.91 in 10,000	-	4	1	1	0	-	-	High	-
[36]	Rezabeigi, D. 2023	Sirjan	Cross-sectional retrospective study	3–5 Days	2019	-	4,816	-	-	-	-	-	2.07 in 10,000	-	5	1	1	0	-	-	High	-
[30]	Habib, A. 2010	Fars	Cross-sectional retrospective study	3–5 Days	2004–2007	-	175,235	88,143	87,091	28	13 (46.4%)	15 (53.5%)	1.6 in 10,000	-	30	28	-	-	-	-	High	The incidence of phenylketonuria in girls and boys was 1.7 in 10000 (95% CI: 1.67–1.72) and 1.5 in 10000 (95% CI: 1.47–1.52), respectively.
[46]	Senemar, S. 2009	Fars	Epidemiological and Clinical Study	Three days	2000–2005	86.6%	70,477	-	-	15 (4 died)	-	-	1 in 4,698	-	-	15 (4 died)	2	13	-	-	High	The prevalence of the disease was 1 in 382 newly born babies in “Eghlid.”
[12]	RezabighiDavarani, F. 2023	Kerman	Cross-sectional and descriptive study	3–5 Days	2012	-	23,841	-	-	4	-	-	1.67 in 10,000	-		4	-	-	-	74.6	High	-
[12]	RezabighiDavarani, F. 2023	Kerman	Cross-sectional and descriptive study	3–5 Days	2013	-	24,562	-	-	1	-	-	0.40 in 10,000	-		1	-	-	-	-	High	-
[12]	RezabighiDavarani, F. 2023	Kerman	Cross-sectional and descriptive study	3–5 Days	2014	-	25,860	-	-	3	-	-	1.16 in 10,000	-		3	-	-	-	-	High	-
[12]	RezabighiDavarani, F. 2023	Kerman	Cross-sectional and descriptive study	3–5 Days	2015	-	26,533	-	-	6	-	-	2.26 in 10,000	-		6	-	-	-	-	High	-
[12]	RezabighiDavarani, F. 2023	Kerman	Cross-sectional and descriptive study	3–5 Days	2016	-	26,518	-	-	7	-	-	2.63 in 10,000	-		7	-	-	-	-	High	-
[12]	RezabighiDavarani, F. 2023	Kerman	Cross-sectional and descriptive study	3–5 Days	2017	-	25,608	-	-	0	-	-	0 in 10,000	-		0	-	-	-	-	High	-
[12]	RezabighiDavarani, F. 2023	Kerman	Cross-sectional and descriptive study	3–5 Days	2018	-	23,446	-	-	2	-	-	0.85 in 10,000	-		2	-	-	-	89.9	High	-
[12]	RezabighiDavarani, F. 2023	Kerman	Cross-sectional and descriptive study	3–5 Days	2019	-	20,146	-	-	2	-	-	0.99 in 10,000	-		2	-	-	-	-	High	-
[34]	Karamifar, H. 2010	Fars	Cross-sectional study	3–5 Days	2007–2008	-	76,966	41,496	35470	8	-	-	-	-	9	8	3	5	100	-	High	The incidence of BH4 deficiency was 1 in 76,966. The incidence of HPA^b^ was 1 in 10,000. The incidence of mild HPA and PKU was 62.5% and 37.5%, respectively.
[13]	Abbaskhanian, A. 2017	Mazandaran	Descriptive-retrospective study	3–5 Days	2007	-	42,328	21,569	20,759	2	0	2	0.47 in 10,000	-	254	2	1	1	-	-	High	-
[13]	Abbaskhanian, A. 2017	Mazandaran	Descriptive-retrospective study	3–5 Days	2008	-	45,703	23,197	22,506	2	2	0	0.44 in 10,000	-	66	2	0	2	-	-	High	-
[13]	Abbaskhanian, A. 2017	Mazandaran	Descriptive-retrospective study	3–5 Days	2009	-	45,442	23,392	22,050	7	2	5	1.54 in 10,000	-	92	7	1	6	-	-	High	-
[13]	Abbaskhanian, A. 2017	Mazandaran	Descriptive-retrospective study	3–5 Days	2010	-	44,420	22,973	21,447	3	0	3	0.68 in 10,000	-	19	3	1	2	-	-	High	-
[13]	Abbaskhanian, A. 2017	Mazandaran	Descriptive-retrospective study	3–5 Days	2011	-	44,342	23,374	21,168	1	0	1	0.23 in 10,000	-	11	1	0	1	-	-	High	-
[13]	Abbaskhanian, A. 2017	Mazandaran	Descriptive-retrospective study	3–5 Days	2012	-	44,352	22,633	21,719	2	1	1	0.45 in 10,000	-	7	2	1	1	-	-	High	-
[13]	Abbaskhanian, A. 2017	Mazandaran	Descriptive-retrospective study	3–5 Days	2013	-	45,994	23,256	22,738	3	3	0	0.65 in 10,000	-	5	3	0	3	-	-	High	-
[13]	Abbaskhanian, A. 2017	Mazandaran	Descriptive-retrospective study	3–5 Days	2014	-	47,220	24,548	22,672	4	3	1	0.85 in 10,000	-	6	4	1	3	-	-	High	-
[13]	Abbaskhanian, A. 2017	Mazandaran	Descriptive-retrospective study	3–5 Days	2015	-	47,443	24,262	23,181	3	2	1	0.63 in 10,000	-	5	3	1	2	-	-	High	-
[33]	Ordooei, M. 2015	Yazd	Descriptive cross-sectional study	3 Days	2010–2011	-	22,131	-	-	4	-	-	-	1/5532 (0.018%)	-	4	1	3	-	-	Moderate	-
[15]	Morovatdar, N. 2015	Khorasan	Epidemiological and Clinical Study	The mean age of diagnosis in the studied subjects was 19 months(ranging from 1 month to 14 years).	2012–2013	80%	69,347	-	-	81 (1 die, 2 no data).	40 (49%)	41 (51%)	57 in 1,000,000	-	-	4	-	-	-	10	High	There were eight families with two PKU patients and one family with 3 PKU patients.
[29]	Saadatpour, Y. 2016	Hormozgan	Cross-sectional study	3–5 Days	2014	-	33,973	-	-	-	-	-	1 in 16,987	-	12	-	-	-	85.70	-	High	-
[29]	Saadatpour, Y. 2016	Hormozgan	Cross-sectional study	3–5 Days	2015	-	37,704	-	-	-	-	-	1 in 37,736	-	3	-	-	-	90.69	-	High	-
[47]	ModaresSadrani, N. 2013	Ardebil	Cross-sectional study	3–5 Days	21 months	85%	44,232	-	-	13	-	-	1 in 3,402	-	-	13	8	5	-	-	Moderate	-
[48]	Mahmoodi, M. 2015	Golestan	Cross-sectional study	3–5 Days	2012–2013	-	74,000	-	-	4	-	-	1 in 18,500	-	32	4	-	-	-	-	Moderate	2 patients were from Gonbad, one from Kalale, and one from Minoodasht.
[28]	Golbahar, J.2002	Shiraz	Descriptive cross-sectional study	-	1996–2001	79%	106, 515	-	-	-	-	-	1 in 3,672	-	-	43	33	10	-	-	Moderate	The total number of cases diagnosed was 43, 29 new cases of PKU were born after January 1, 1996, so they calculated the incidence of PKU as 1 in 3,672.
[49]	Moradi, K. 2014	Firozabad ward	Descriptive-analytical study	--	2013	-	31,520	-	-	10	5	5	-	10 in 31520	-	10	9	1	-	-	High	-
[37]	Saadatinasab, Z. 2015	Birjand	Retrospective descriptive	3–5 days	2012–2014	100%	12,293	-	-	1	-	-	0.813 in 10,000	-	21	1	-	-	100	-	High	-
[37]	Saadatinasab, Z. 2015	Qaen	Retrospective descriptive	3–5 days	2012–2014	100%	5,434	-	-	2	-	-	3.68 in 10,000	-	17	2	-	-	100	-	High	-
[37]	Saadatinasab, Z. 2015	Darmian	Retrospective descriptive	3–5 days	2012–2014	100%	2,215	-	-	0	-	-	0 in 10,000	-	5	0	-	-	100	-	High	-
[37]	Saadatinasab, Z. 2015	Sarbisheh	Retrospective descriptive	3–5 days	2012–2014	100%	1,551	-	-	0	-	-	0 in 10,000	-	8	0	-	-	100	-	High	-
[37]	Saadatinasab, Z. 2015	Sarayan	Retrospective descriptive	3–5 days	2012–2014	100%	1,206	-	-	0	-	-	0 in 10,000	-	0	0	-	-	100	-	High	-
[37]	Saadatinasab, Z. 2015	Tabas	Retrospective descriptive	3–5 days	2012–2014	100%	2,180	-	-	0	-	-	0 in 10,000	-	1	0	-	-	100	-	High	-
[37]	Saadatinasab, Z. 2015	Ferdows	Retrospective descriptive	3–5 days	2012–2014	100%	1,937	-	-	0	-	-	0 in 10,000	-	1	0	-	-	100	-	High	-
[37]	Saadatinasab, Z. 2015	Nehbandan	Retrospective descriptive	3–5 days	2012–2014	100%	2,302	-	-	0	-	-	0 in 10,000	-	1	0	-	-	100	-	High	-
[37]	Saadatinasab, Z. 2015	Boshruyeh	Retrospective descriptive	3–5 days	2012–2014	100%	958	-	-	0	-	-	0 in 10,000	-	1	0	-	-	100	-	High	-
[37]	Saadatinasab, Z. 2015	Zirkuh	Retrospective descriptive	3–5 days	2012–2014	100%	151	-	-	0	-	-	0 in 10,000	-	1	0	-	-	100	-	High	-
[37]	Saadatinasab, Z. 2015	Khosf	Retrospective descriptive	3–5 days	2012–2014	100%	86	-	-	0	-	-	0 in 10,000	-	0	0	-	-	100	-	High	-
[50]	Badiee, M. 2014	Torbat-E- Heydarieh	Descriptive analytical study	3–5 day	2011–2013	-	11,091	5,701	5,390	3	-	-	1 in 3,697	-	16	3	1	2	-	-	High	Of the 16 infants found at screening, 15 had screen-positive cases and one classical PKU.
[51]	Ajami, A. 2014	Isfahan	Cross-sectional study	-	2012–2013	-	77,000	-	-	45	-	-	-	1 in 6,500	-	45	12	33	-	-	High	-
[38]	Ganji, F. 2018	Chaharmahal and Bakhtiari	Descriptive-analytical study	3–5 days	2012	-	13,022	-	-	1	-	-	1 in 13,022	-	-	1	-	-		80	High	
[38]	Ganji, F. 2018	Chaharmahal and Bakhtiari	Descriptive-analytical study	3–5 days	2013	-	19,612	-	-	4	-	-	4 in 19,612	-	-	4	-	-		81	High	
[38]	Ganji, F. 2018	Chaharmahal and Bakhtiari	Descriptive-analytical study	3–5 days	2014	-	19,753	-	-	3	-	-	3 in 19,753	-	-	3	-	-		83	High	
[38]	Ganji, F. 2018	Chaharmahal and Bakhtiari	Descriptive-analytical study	3–5 days	2015	-	20,893	-	-	3	-	-	3 in 20,893	-	--	3	-	-	-	84.6	High	-
[52]	Behineh, M. 2018	Khonj city (Fars)	Cross-sectional study	3–5 days	2007–2014	-	6,399	-	-	2	1	1	-	2 in 6,399	-	2	-	-	-	-	High	-
[53]	Soori, M. 2018	Nahavand	Cross-sectional (descriptive-analytical) study	3–5 days	2016–2018	-	5,704	-	-	0	-	-	-	0	0	0	0	0	-	78.8	High	-

^a^ Phenylketonuria

^b^ Hyperphenylalaninemia

*Some studies were considered more than one because they were investigated in different populations over many years. These studies estimated prevalence for over a year in different regions

### Prevalence of screen-positive cases

**Fig. 2:**
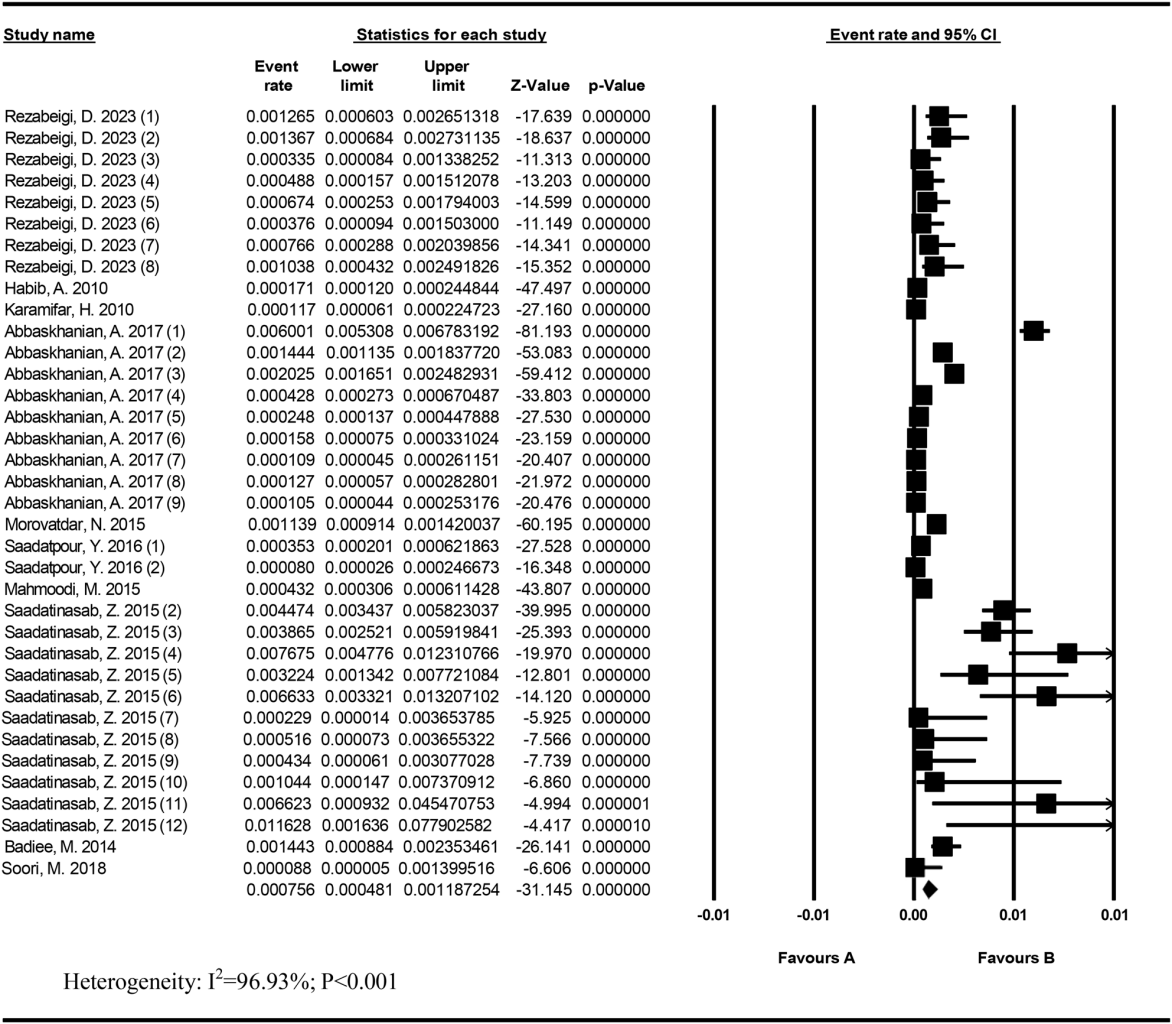
Prevalence of screen-positive cases in Iran

Heterogeneity was high for these studies (I^2^ = 96.93%; *P* < 0.001). The prevalence of screen-positive cases in 262,132 Iranian neonates was 75.6/100,000 (95% CI: 48.1–118.72). The lowest prevalence was related to Saadatpour, Y. (2015) (8/100,000) in Hormozgan, and the highest prevalence was Saadatinasab et al. in 2012–2014 in Khosf (1162.8/100,000) (Fig. 2).

#### Subgroup analysis of the prevalence of screen-positive cases based on region, province, and quality of studies

Subgroup analysis of the prevalence of screen-positive cases based on four geographical regions (*P* < 0.001), provinces (*P* < 0.001), and quality of studies (*P* = 0.04) in Iran showed significant differences (Tables 2a). The prevalence of screen-positive cases from the most to least is 263.8/100,000 (95% CI: 152.2–457) in East, 44.3/100,000 (95% CI: 18.6-105.6) in North, 42.4/100,000 (95% CI: 24.0-74.7)in South and 8.8/100,000 (95% CI: 0.5–140.0)in West of Iran, respectively.

**Table 2:** Subgroup analysis based on region, province, and quality of studies

Variable		Studies (*N*)*		Sample (*N*)		Heterogeneity			95% CI	Pooled (Per 100,000)
				Patient	All	I^2^	*P*-Value			
**(A) Screen-positive cases**										
Region	East	13		206	110,751	89.84	< 0.001		152.2–457	263.8
	North	10		497	481,244	98.59	< 0.001		18.6-105.6	44.3
	South	12		89	368,669	84.16	< 0.001		24.0-74.7	42.4
	West	1		0	5,704	-	-		0.5–140	8.8
	Test for subgroup differences: Q = 26.71, df(Q) = 3, *P* < 0.001									
Provinces	Kerman	8		35	44,791	4.51	0.39		61.7-122.1	86.8
	Fars	2		39	252,201	0.61	0.31		11.4–21.5	15.7
	Golestan	1		32	74,000	-	-		30.6–61.1	43.2
	Hamedan	1		0	5,704	-	-		0.5–140	8.8
	Hormozgan	2		15	71,677	81.24	0.02		4.3–77.8	18.2
	Mazandaran	9		465	407,244	98.52	< 0.001		18.2-109.3	44.6
	Razavi Khorasan	1		95	80,438	-	-		97.0-144.9	118.5
	South Khorasan	8		111	30,313	59.91	< 0.01		258.3–583.0	388.1
	Test for subgroup differences: Q = 194.57, df(Q) = 7, *P* < 0.001									
Quality	Medium	1		32	74,000	-	-		30.6–61.1	43.2
	High	35		760	892,368	96.81	< 0.001		48.8-121.5	77.0
	Test for subgroup differences: Q = 3.89, df(Q) = 1, *P* = 0.04									
**(B)** **Confirmed PKU**^**a**^										
Region	Center	6	60		172,411	74.08		0.002	10.2–44.6	21.3
	East	13	13		110,751	41.97		0.055	16.0-64.4	32.1
	North	11	44		525,476	60.51		0.005	4.7–13.1	7.9
	South	22	131		758,116	55.47		0.001	12.0-23.1	16.7
	West	13	84		434,878	18.49		0.257	16.0-25.9	20.3
	Test for subgroup differences: Q = 14.28, df(Q) = 4, *P* = 0.006									
Provinces	Kerman	16	32		241,305	0		0.642	12.6–24.7	17.6
	Ardebil	1	13		44,232	-		-	17.1–50.6	29.4
	Chaharmahal and Bakhtiari	4	11		73,280	0		0.846	8.7–28.3	15.6
	Fars	4	53		329,077	14.80		0.318	12.3–22.7	16.7
	Golestan	1	4		74,000	-		-	2.0-14.4	5.4
	Hamedan	1	0		5,704	-		-	0.5–140	8.8
	Hormozgan	1	3		81,219	-		-	1.2–11.5	3.7
	Isfahan	1	45		77,000	-		-	43.6–78.3	58.4
	Kermanshah	1	10		31,520	-		-	17.1–59.0	31.7
	Lorestan	11	74		397,654	20.10		0.252	14.9–25.1	19.3
	Mazandaran	9	27		407,244	0		0.555	5.2–11.0	7.5
	Razavi Khorasan	2	7		80,438	75.57		0.043	2.7–55.2	12.2
	Shiraz	1	43		106,515	-		-	29.9–54.4	40.4
	South Khorasan	11	6		30,313	0		0.549	23.8–82.0	44.1
	Yazd	1	4		22,131	-		-	6.8–48.1	18.1
	Test for subgroup differences: Q = 122.35, df(Q) = 14, *P* < 0.001									
Quality	Medium	4	64		246,878	59.75		0.001	10.0-42.9	20.7
	High	61	268		1,754,754	81.67		< 0.001	12.9–19.9	16.0
	Test for subgroup differences: Q = 0.447, df(Q) = 1, *P* = 0.504									
**(C) Hyperphenylalaninemia (HPA)**										
Region	Center	2	36		99,131	75.56		0.056	9.2–82.4	27.5
	East	1	2		11,091	-		-	4.5–72.1	18.0
	North	10	26		451,476	0		0.466	4.7–10.1	6.9
	South	12	31		379,968	0		0.538	7.7–15.0	10.8
	West	2	1		37,224	0		0.557	0.9–22.1	4.5
	Test for subgroup differences: Q = 8.54, df(Q) = 4, *P* = 0.074									
Provinces	Kerman	8	1		44,791	-		-	4.1–26.1	10.4
	Ardebil	1	5		44,232	-		-	4.7–27.2	11.3
	Fars	2	18		147,443	74.57		0.047	4.2–32.1	11.6
	Hamedan	1	0		5,704	-		-	0.5–140	8.8
	Hormozgan	1	2		81,219	-		-	0.6–9.8	2.5
	Isfahan	1	33		77,000	-		-	30.5–60.3	42.9
	Kermanshah	1	1		31,520	-		-	0.4–22.5	3.2
	Mazandaran	9	21		407,244	0		0.520	4.0-9.4	6.1
	Razavi Khorasan	1	2		11,091	-		-	4.5–72.1	18.0
	Shiraz	1	10		106,515	-		-	5.1–17.4	9.4
	Yazd	1	3		22,131	-		-	4.4–42.0	13.6
	Test for subgroup differences: Q = 65.97, df(Q) = 10, *P* < 0.001									
Quality	Medium	3	18		172,878	0		0.840	6.6–16.7	10.5
	High	24	78		806,012	68.91		< 0.001	4.7–12.6	7.7
	Test for subgroup differences: Q = 0.815, df(Q) = 1, *P* = 0.367									
**(D) Classical PKU or Severe PKU**										
Region	Center	2	13		99,131	29.33		0.234	4.6–32.1	12.2
	East	1	1		11,091	-		-	1.3–64.0	9.0
	North	10	8		44,232	55.02		0.018	9.0-36.2	18.1
	South	12	45		379,968	67.15		< 0.001	5.0-23.2	10.8
	West	2	9		37,224	0		0.416	14.2–50.7	26.8
	Test for subgroup differences: Q = 16.56, df(Q) = 4, *P* = 0.002									
Provinces	Kerman	8	6		44,791	0		0.787	11.3–45.1	22.6
	Ardebil	1	8		44,232	-		-	9.0-36.2	18.1
	Fars	2	5		147,443	0		0.728	1.4–8.2	3.4
	Hamedan	1	0		5,704	-		-	0.5–140	8.8
	Hormozgan	1	1		81,219	-		-	0.2–8.7	1.2
	Isfahan	1	12		77,000	-		-	8.9–27.4	15.6
	Kermanshah	1	9		31,520	-		-	14.9–54.9	28.6
	Mazandaran	9	6		407,244	-		-	0.9–3.9	1.9
	Razavi Khorasan	1	1		11,091	-		-	1.3–64.0	9.0
	Shiraz	1	33		106,515	-		-	22.0-43.6	31.0
	Yazd	1	1		22,131	-		-	0.6–32.1	4.5
	Test for subgroup differences: Q = 72.60, df(Q) = 10, *P* < 0.001									
Quality	Medium	3	33		806,012	60.78		0.078	22.0-43.6	31
	High	24	40		106,515	56.32		< 0.001	3.7–10.6	6.2
	Test for subgroup differences: Q = 7.22, df(Q) = 1, *P* = 0.007									

N: Number; CI: confidence interval

^a^ Phenylketonuria

*Some studies were considered more than one because they were investigated in different populations over many years. These studies estimated prevalence for over a year in different regions

### Prevalence of confirmed PKU

**Fig. 3:**
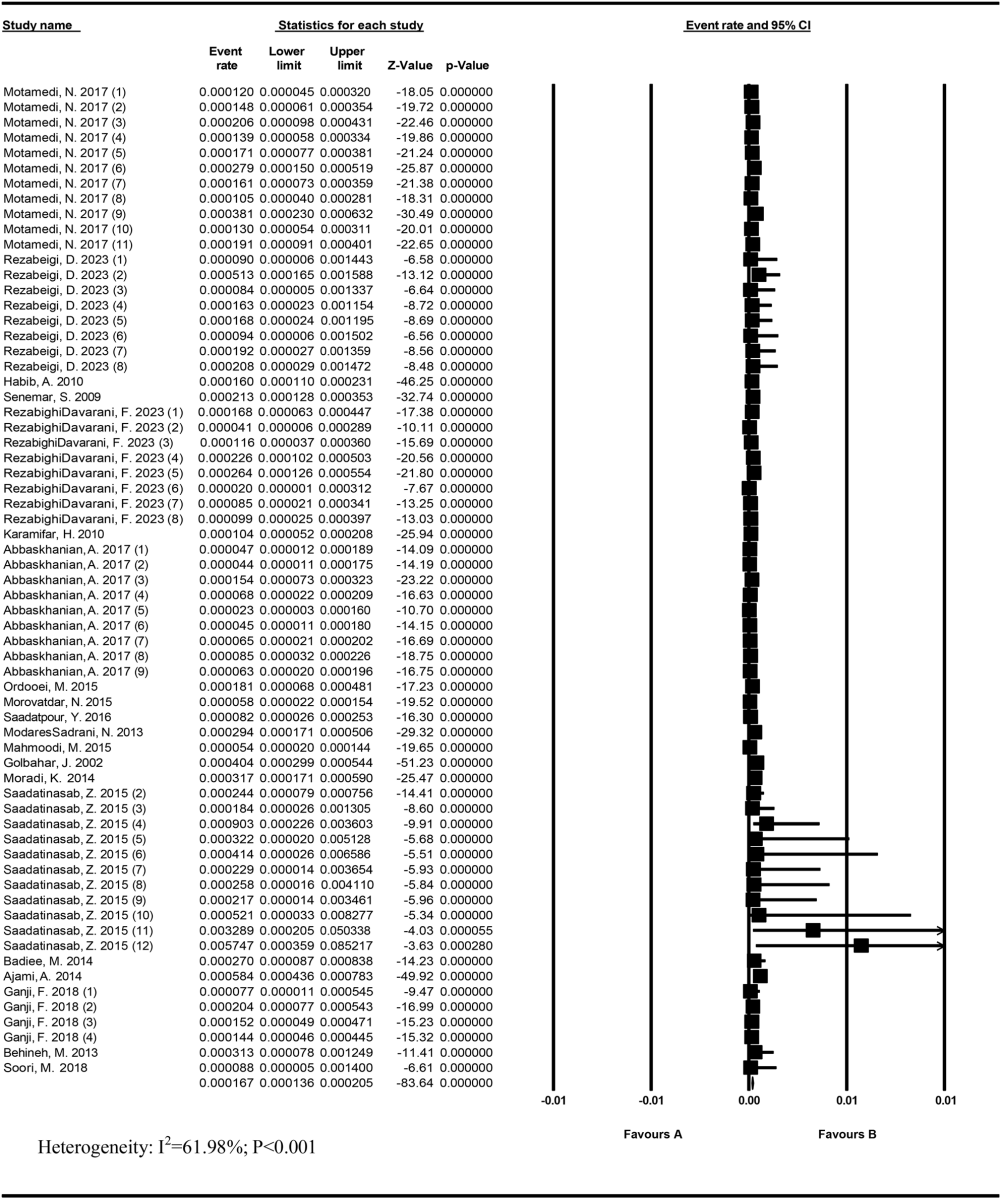
Prevalence of confirmed PKU in Iran

Heterogeneity was high for the studies (I^2^ = 61.98%; *P* < 0.001). The prevalence of confirmed PKU in 1,992,090 Iranian neonates was 16.7/100,000 (95% CI: 13.6–20.5). The lowest and highest prevalence was related to Rezabeigi Davarani, F. (2017) in Kerman (2/100,000) and Saadatinasab et al. in 2012–2014 in Khosf (574.7/100,000), respectively (Fig. 3).

#### Subgroup analysis of confirmed PKU prevalence based on region, province, and quality of studies

The prevalence of confirmed PKU in Center, East, North, South and West of Iran was 21.3/100,000 (95% CI: 10.2–44.6), 32.1/100,000 (95% CI: 16.0-64.4), 7.9/100,000 (95% CI: 4.7–13.1), 16.7/100,000 (95% CI: 12.0-23.1) and 20.3/100,000 (95% CI: 16.0-25.9), respectively, and the differences in subgroup analysis were significant (*P* = 0.006) (Tables 2b).

In subgroup analysis based on province, the lowest and highest prevalence of confirmed PKU was in Hormozgan (3.7/100,000) and Isfahan (58.4/100,000) provinces, respectively, and the difference was significant (*P* < 0.001) (Tables 2b).

In subgroup analysis based on the quality of studies, the prevalence of confirmed PKU in moderate and high-quality studies was 20.7/100,000 (95% CI: 10.0-42.85) and 16.0/100,000 (95% CI: 12.9–19.9), respectively, and the differences in subgroup analysis were not significant (*P* = 0.504) (Tables 2b).

### The prevalence of different types of confirmed PKU

**Fig. 4:**
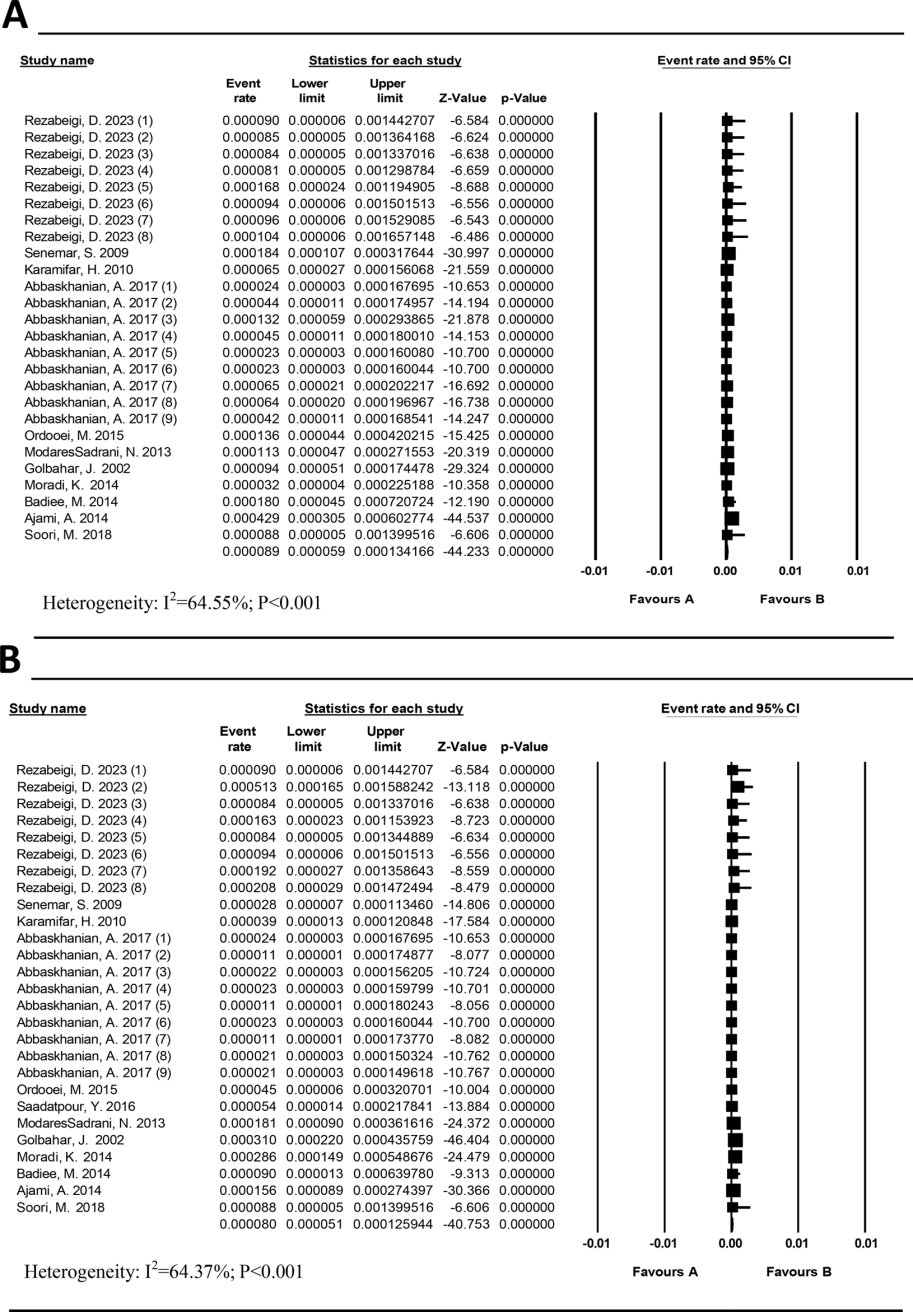
Prevalence of Hyperphenylalaninemia (HPA) (**a**) Classical PKU (**b**) in Iran

The heterogeneity of HPA and classical PKU studies was moderate (I^2^ = 64.55%; *P* < 0.001) and (I^2^ = 64.37%; *P* < 0.001), respectively. The prevalence of HPA (Fig. 4a) and classical PKU (Fig. 4b) was estimated at 8.9/100,000 (95% CI: 5.9–13.41) and 8.0/100,000 (95% CI: 5.1–12.59), respectively.

#### Subgroup analysis of HPA prevalence based on region, province, and quality of studies

Subgroup analysis of the prevalence of HPA based on provinces showed significant differences (*P* < 0.001), but it was not significant based on geographical regions and quality (*P* = 0.074 and *P* = 0.367), respectively (Table 2c).

#### Subgroup analysis of classical PKU prevalence based on region, province, and quality of studies

Subgroup analysis of the prevalence of classical PKU showed significant differences based on five geographical regions (*P* = 0.002), provinces *P* < 0.001), and quality of studies (*P* = 0.007) (Table 2d).

### Prevalence of PKU based on gender

**Fig. 5:**
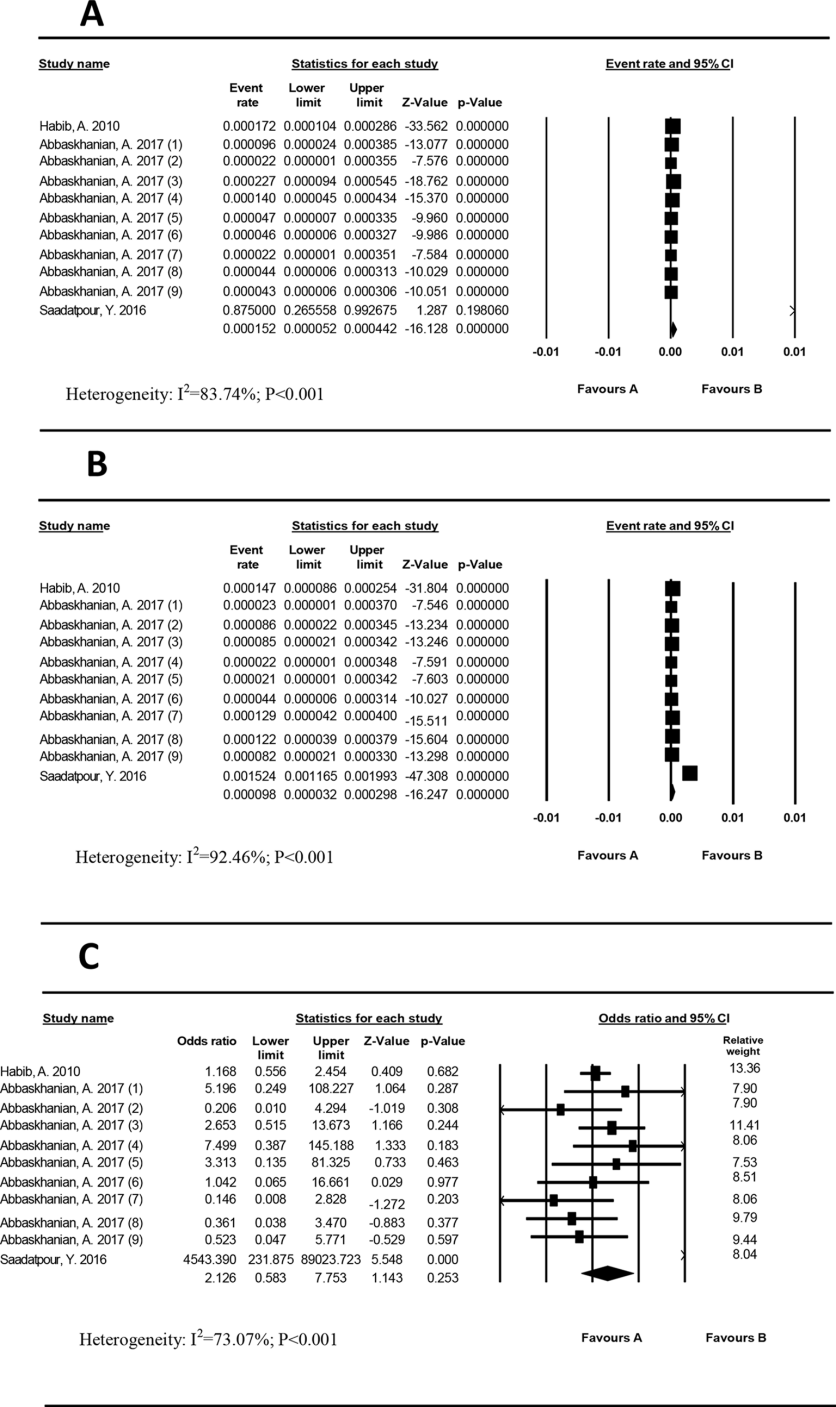
Prevalence of phenylketonuria in girls (**a**) and boys (**b**) and girls to boys odds ratio (**d**) in Iran

The heterogeneity of PKU studies in girls and boys was high (I^2^ = 83.74%; *P* < 0.001) and (I^2^ = 92.46%; *P* < 0.001), respectively. The prevalence of PKU in girls (Fig. 5a) and boys (Fig. 5b) was estimated to be 15.2/100,000 (95% CI: 5.2–44.2) and 9.8/100,000 (95% CI: 3.2– 29.8), respectively. Heterogeneity was also estimated for girls-boys studies (I2 = 73.07%; *P* < 0.001). The girls-boys OR of PKU prevalence was insignificant (OR = 2.126 (95% CI: 0.58–7.75, *P* = 0.253) (Fig. 5c).

### Meta-regression

Meta-regression for the prevalence of screen-positive cases, confirmed PKU, classical PKU, and HPA based on the year of study was (Meta-regression coefficient: -0.061, 95% CI -0.21 to 0.086, *P* = 0.414), (Meta-regression coefficient: -0.021, 95% CI -0.067 to 0.023, *P* = 0.82), (Meta-regression coefficient: 0.003, 95% CI -0.079 to 0.086, *P* = 0.648), and (Meta-regression coefficient: -0.013, 95% CI − 0.091 to 0.064, *P* = 0.986), respectively (Additional File 1: Fig. 6).

Moreover, the meta-regression model for the prevalence of PKU based on the year was also studied in girls (Meta-regression coefficient: 0.136, 95% CI -0.237 to 0.51, *P* = 0.008), and boys (Meta-regression coefficient: 0.098, 95% CI -0.222 to 0.418, *P* = 0.907), and it was increasing significantly in girls (Additional File 2: Fig. 7).

### Sensitivity analysis

Sensitivity analysis for the prevalence of screen-positive cases (Additional File 3: Fig. 8), confirmed PKU (Additional File 4: Fig. 9), classical PKU (Additional File 5: Fig. 10a), and HPA (Additional File 5: Fig. 10b) showed that the pooled estimate was robust. The sensitivity analysis of PKU in girls and boys is also shown in Additional File 6: Fig. 11.

### Publication bias

The publication bias based on Egger’s and Begg’s tests for the prevalence of screen-positive cases (*P* = 0.001 and *P* = 0.47, respectively), confirmed PKU (*P* < 0.001 and *P* = 0.28), HPA (*P* < 0.001 and *P* = 0.27), and classical PKU (*P* < 0.001 and *P* = 0.23) was significant based on Egger’s test but not Begg’s tests (Additional File 7: Fig. 12).

According to Egger and Begg’s tests, publication bias for girls, boys, and girls-boys showed in Additional File 8: Fig. 13 (*P* = 0.96 and *P* = 0.16 for girls), (*P* = 0.001 and *P* = 0.042 for boys), (*P* = 0.52 and *P* = 0.43 for girls-boys).

## Discussion

The previous systematic review study was based on articles before 2019 investigating the epidemiology of PKU screening in Iranian infants. However, several previous studies were not included. Since then, several extensive, rigorously designed studies have provided updated data. As a result of the importance of PKU and its high financial and personal burden on Iranians, this systematic review was conducted to provide a general picture of its prevalence in Iran. In this study, 20 Persian and English-published articles on the prevalence of PKU in Iran were reviewed. Based on these studies, the prevalence of screen-positive cases in 262,132 Iranian neonates was 75.6/100,000, however confirmed PKU was calculated at 16.7 per 100,000 Iranian neonates. In the NBS program, the Phe level above 4 mg/dl (even in some cases above 2 mg/dl) is considered a case of PKU and requires HPLC confirmatory tests. This cutoff level increases the sensitivity of the screening test and reduces the risk of missing the diagnosis of phenylalanine metabolism disorders in individuals with less pronounced Phe‑circulating levels at the first screening. This may be one reason for the large difference between the prevalence of disease in the confirmed PKU cases and the screen-positive cases.

In conclusion, the number of patients identified in the initial screening using Guthrie cards (Fluorometric or Enzymatic method) is usually higher than confirmed by the HPLC method. This discrepancy may be due to the following reasons: (1) High sensitivity and low specificity of the Guthrie method: The Guthrie method is designed for screening and has high sensitivity, meaning it aims to detect all possible cases. However, it has lower specificity, which can lead to false positives-healthy individuals may be incorrectly flagged as affected. (2) Interfering factors during initial sample collection: Factors such as the time of sampling, the infant’s diet, low birth weight, or prematurity can falsely elevate phenylalanine levels in the initial test. This may result in some newborns being mistakenly identified as at risk for PKU. (3) HPLC is more accurate: HPLC offers higher specificity and precision, allowing for differentiation between various forms of phenylalanine metabolism disorders (e.g., mild HPA vs. classical PKU). Therefore, many initially positive cases are later found to be false positives. (4) The goal of screening is to avoid missing true cases: The primary goal of initial screening is to identify all potential cases, even at the cost of some false positives. Final confirmation must always be done using more accurate methods such as HPLC or tandem mass spectrometry (MS/MS). Diagnosing the disease based on clinical symptoms at birth is impossible. Therefore, all babies should be screened between the 3rd and 5th day after birth based on NBS [40]. NBS is a successful public health system used for the early detection of hazardous hereditary diseases. A failure to diagnose these diseases in time can lead to mental retardation, permanent disability, and even death [41]. NBS was developed for PAH deficiency in North America and England in 1960 and other developed countries in 1970 [42]. The NBS has resulted in significant savings for society and benefits for the affected people because almost all cases of PAH deficiency are identified following screening tests. The first pilot study to evaluate HPA in infants in Iran was started in 1982 in Tehran. Iran’s first national newborn screening program in Fars province in 2004 and then in Mazandaran province in 2007. According to the law, all Iranian babies must be screened since 2015 [13].

Based on this study, the prevalence of screen-positive cases in 262,132 Iranian neonates was estimated to be 75.6/100,000, but in one recent meta-analysis in 873,174 Iranian neonates was 45.6/100,000 [11]. At first glance, this increase can be considered worrying, examining the studies included in this meta-analysis published in 2019, the reason for this increase in prevalence can be attributed to the difference in its inclusion criteria with the present study. Also, the previous study unfortunately did not consider all articles and abstracts published in Persian congresses and journals, which could reduce the accuracy of the prevalence study and, as a result, justify the lower reporting of the disease prevalence. It also seems that there has been more attention and care recently paid to the NBS program, which has led to more newborn screening and, as a result, an increase in diagnosed patients.

As we mentioned above, the prevalence of confirmed PKU was estimated to 16.7 per 100,000 Iranian neonates. Based on Shokri et al. [11], the prevalence of PKU in Iran was calculated at 16.5/100,000, which was lower than our study. Several previous studies were not included, which may explain why they underreported the prevalence of PKU up to 2019. However, this prevalence was expected to be less than this increase with increased premarital education, counseling, and people’s information level. PKU prevalence was estimated in 59 countries by a recent meta-analysis study, with Thailand having the lowest prevalence at 0.44/100,000 and Italy having the highest prevalence at 27.37/100,000 [22]. However, data on the prevalence of PKU in some countries (such as India or Finland) were outdated. In another meta-analysis of 46 studies from 1964 to 2017, the highest and lowest prevalences were in Turkey (38.13 per 100,000) and Thailand (0.3 per 10,000), respectively [3]. In Foremanin et al. [43], the global prevalence of PAH deficiency was estimated at 6.4 per 100,000 births (from 0.3 per 100,000 births in Southeast Asia to 11.8 per 100,000 births in the Middle East/North Africa). Hence, based on the global reports mentioned above, the prevalence reported in our study is significant for Iran and PKU affects a significant number of Iranian children who require specific treatments and management throughout their lives, and this high prevalence and the need for many of them to have a Phe-free lifelong diet double the importance of addressing this group. On the other hand, PKU patients require a Phe-free formula that is costly and sometimes difficult to provide, and with the high prevalence, the need to sustainably meet these needs at the national level increases.

Confirmed cases of PKU were divided into two subgroups: HPA (A prevalence of 8.9/100,000) and classical PKU (A prevalence of 8/100,000), this indicates a lower number of people with the classic type of PKU than the mild and moderate and HPA types, which is good because patients with milder PKU are more likely to respond to BH₄ (sapropterin) therapy, while only about 8% of classic PKU patients show responsiveness [44]. Classical PKU is associated with more severe symptoms, including brain damage, seizures, and behavioral disorders. In contrast, milder forms generally present a lower risk of neurological damage and may not require strict treatment. Even, patients with classical PKU typically require lifelong dietary treatment, whereas those with milder variants may not need intensive or long-term therapy. Therefore, understanding the distribution and severity of PKU subtypes is essential for personalized treatment planning and future research. The prevalence of HPA and classical PKU was estimated at 9.7 per 100,000 and 4.4 per 100,000, respectively by Shokri et al. [11]. The prevalence of classical PKU based on a meta-analysis based on articles until 2018, showed that the global prevalence of classical PKU was 6.002 per 100,000 neonates, and the highest prevalence was seen in Eastern Mediterranean (9.83 per 100,000 neonates) [3]. This information and data places Iran among the countries with the highest prevalence of PKU in the world. This could be a warning sign for the genetic health of the country’s population.

Similar to another meta-analysis study in Iran, this study showed that the prevalence of PKU in girls (15.2/100,000) was estimated to be higher than in boys (9.8/100,000). Compared to the mentioned meta-analysis study, the prevalence of PKU was estimated to be higher in girls (15.2/100,000 against 13.3/100,000) but lower in boys (9.8/100,000 against 10.9/100,000). However, it can be said that since this disease is considered an autosomal recessive genetic disorder and the defective gene is located on one of the non-sex chromosomes (autosomes), it is transmitted equally to both sexes. The ratio of girls to boys has been reported to be approximately 1:1 in most studies, and these minor statistical differences in the studies mentioned above can be attributed to sampling error, random factors, sample size, or regional differences.

PKU is inherited from the babies’ parents because it is transmitted in a recessive pattern, meaning both parents should share a mutated version of the PAH gene for a child to develop PKU. When both parents have PKU, their child would also have PKU, and the problem is more common among consanguineous marriages [45], which can be one of the reasons for the high prevalence of PKU in Iran. Especially despite premarital counseling and necessary training, consanguineous marriage is prevalent among Iranians, and based on our review, even some families had two or more PKU children. Despite the great importance of this issue for analysis and conclusions, unfortunately, only 25% of the articles included in this study mentioned the rate of consanguineous marriage.

### Limitations

A major drawback of prevalence studies is that they use retrospective data from medical records or registries; in the case of our subject, because NBS is so essential at birth, the relevant organization accurately records the data. One of the other limitations of our study was the insensitivity of internal databases to “AND” and “OR” operators to search for the combination. The appropriate search strategy for each database was used to avoid missing data and resolve this issue. Examining the genotype of this disease was not our aim in this study, so we did not determine it. Also, unfortunately, its genotype was not mentioned in the articles. We hope that this will be investigated in future studies. Also, due to that various studies had not categorized the number of patients with Phe levels less than 20 mg/dl in a specific way, so we had to classify Phe levels above 20 mg/dl as severe or classical PKU and Phe levels less than 20 mg/dl as HPA. Examining the prevalence of PAH-related PKU vs. BH4-related would provide valuable information and it can provide a more comprehensive and complete view for readers, the included articles did not specify the PAH-related PKU vs. BH4-related group, so there was no precise information for data extraction.

### Strengths

This search was in publications in English and Persian to find all articles in Iran. A comprehensive search strategy was used to maximize the identification of all relevant articles and even grey literature. The random effects model was used in significant heterogeneity studies, and subgroup analysis and meta-regression models were utilized to find the cause of heterogeneity and publication bias. The present meta-analysis can be generalized to the entire population due to the exclusion of studies on specific patients (such as mentally retarded patients), studies with non-random samples, and also the sample size related to non-Iranian people (such as Afghans). In addition, the studies’ authors were contacted to resolve the suspicious cases, to decide on duplicate articles, and to get the full text of some articles.

## Conclusions

In this systematic review and meta-analysis, the prevalence of confirmed PKU in Iran was 16.7 per 100,000 Iranian neonates. Iran has a significant prevalence of this disease, according to Iranian and global studies mentioned above. Iran has a high prevalence of PKU, a disease that has high complications for children, and the ability to control the disease with a Phe-restricted diet as well as special Phe-free formulas for these patients. Keeping this population in mind and reducing its prevalence is vital. Therefore, conducting an extensive study in the form of a biobank to investigate the prevalence and incidence of PKU in Iran, as well as to investigate the deficiencies and problems of these patients for management and treatment, as well as to control its prevalence and incidence, is of great scientific importance. Moreover, the importance of pre-marital counseling and education more seriously should be considered due to the nature of disease transmission from parents to children.

## Supplementary Information

Below is the link to the electronic supplementary material.

Supplementary Material 1: Additional File 1: Fig. 6 Meta-regression model for prevalence of Screen-positive cases (a), Confirmed PKU (b), Classical PKU (c), and HPA (d) based on the year of study

Supplementary Material 2: Additional File 2: Fig. 7 Meta-regression model for prevalence of PKU in girls (a) and PKU in boys (b) based on year of study

Supplementary Material 3: Additional File 3: Fig. 8 Sensitivity analysis of the prevalence of screen-positive cases in neonatal screening programs in Iran

Supplementary Material 4: Additional File 4: Fig. 9 Sensitivity analysis of the prevalence of confirmed PKU in neonatal screening programs in Iran

Supplementary Material 5: Additional File 5: Fig. 10 Sensitivity analysis of the prevalence of Classic PKU (a) HPA (b) in neonatal screening programs in Iran

Supplementary Material 6: Additional File 6: Fig. 11 Sensitivity analysis of the prevalence of confirmed PKU in girls (a) and boys (b) in neonatal screening programs in Iran

Supplementary Material 7: Additional File 7: Fig. 12 Publication bias for the prevalence of Screen-positive cases (a), Confirmed PKU (b), Classical PKU (c), and HPA (d) based on the year of study

Supplementary Material 8: Additional File 8: Fig. 13 Publication bias for prevalence of confirmed PKU in girls (a), confirmed PKU in boys (b), and girls to boys odds ratio (c) based on year of study

## Data Availability

Data sets supporting the conclusions of this study are included in this published manuscript and its Supplementary Information. All articles are included by search criteria and full publication details are provided. Further questions can be directed to the corresponding author.

## Abbreviations

BH4: Tetrahydrobiopterin
CMA: Comprehensive Meta-analysis software
HPA: Hyperphenylalaninemia
HPLC: High-Performance Liquid Chromatography method
IEM: Inborn Errors of Metabolism
IranDoc: Iranian Research Institute for Information Science and Technology
MOOSE: Meta-analyses Of Observational Studies in Epidemiology
NBS: Newborn screening
NOS: Newcastle-Ottawa Scale
OR: Odds ratio
PAH: Phenylalanine hydroxylase
PICO: Population, Intervention, Comparison, Outcomes
PKU: Phenylketonuria
PRISMA: Preferred Reporting Items for Systematic Reviews and Meta-analysis
PROSPERO: International prospective register of systematic reviews
SID: Scientific Information Database
