# Supplementary material for: The prevalence of phenylketonuria (PKU) and hyperphenylalaninemia (HPA) in Iran: a systematic review and meta-analysis
Source: Orphanet J Rare Dis. 2026 Feb 25;21:146. doi: 10.1186/s13023-026-04255-z (PMC13067558; doi:10.1186/s13023-026-04255-z)
Supplement: Supplementary file 1 — Supplementary Material 1: Additional File 1: Fig. 6 Meta-regression model for prevalence of Screen-positive cases (a), Confirmed PKU (b), Classical PKU (c), and HPA (d) based on the year of study [file 13023_2026_4255_MOESM1_ESM.pdf]

A

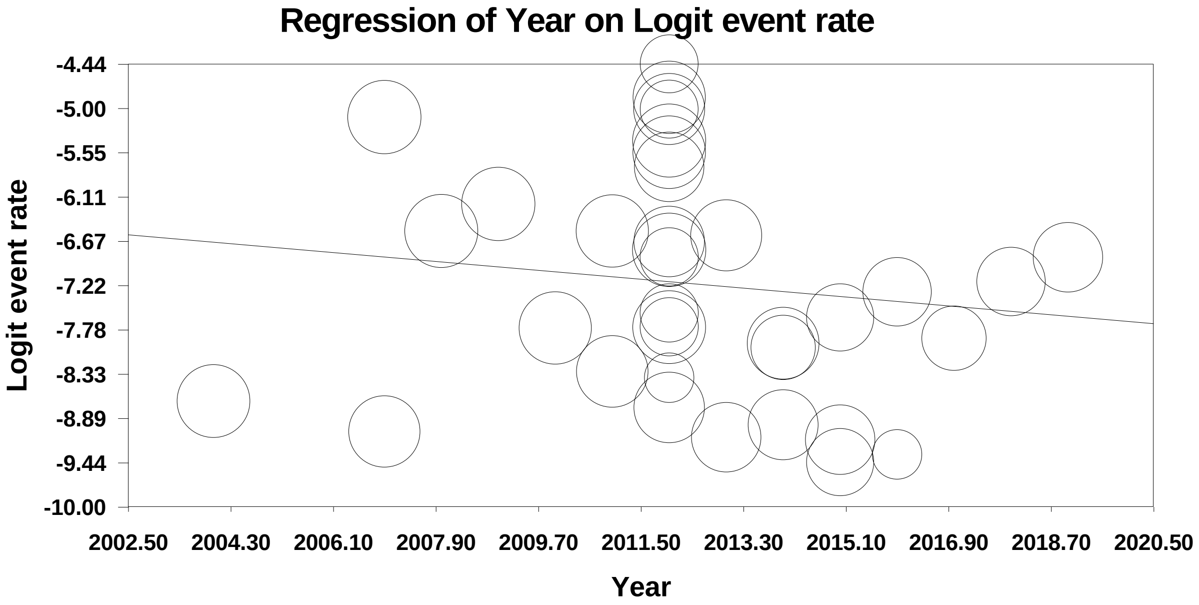

Meta-regression coefficient: - 0.061, 95% CI −0. 21 to 0.086, P=0.414

B

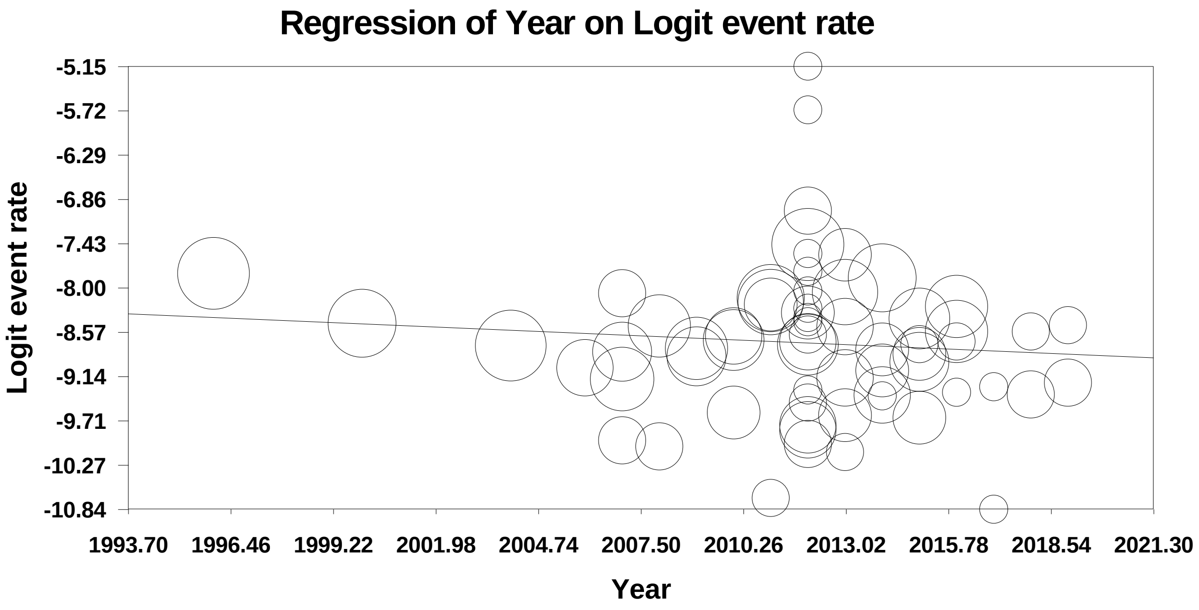

Meta-regression coefficient: -0.021, 95% CI −0.067 to 0.023, P=0.82

C

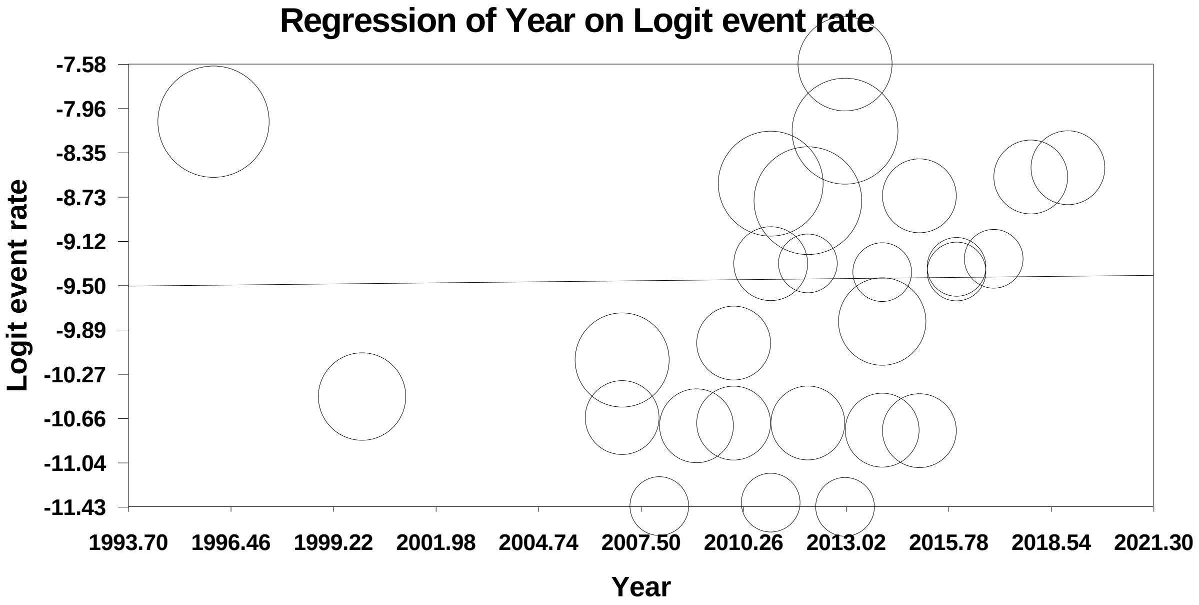

Meta-regression coefficient: 0.003, 95% CI -0.079 to 0.086, P=0.648

D

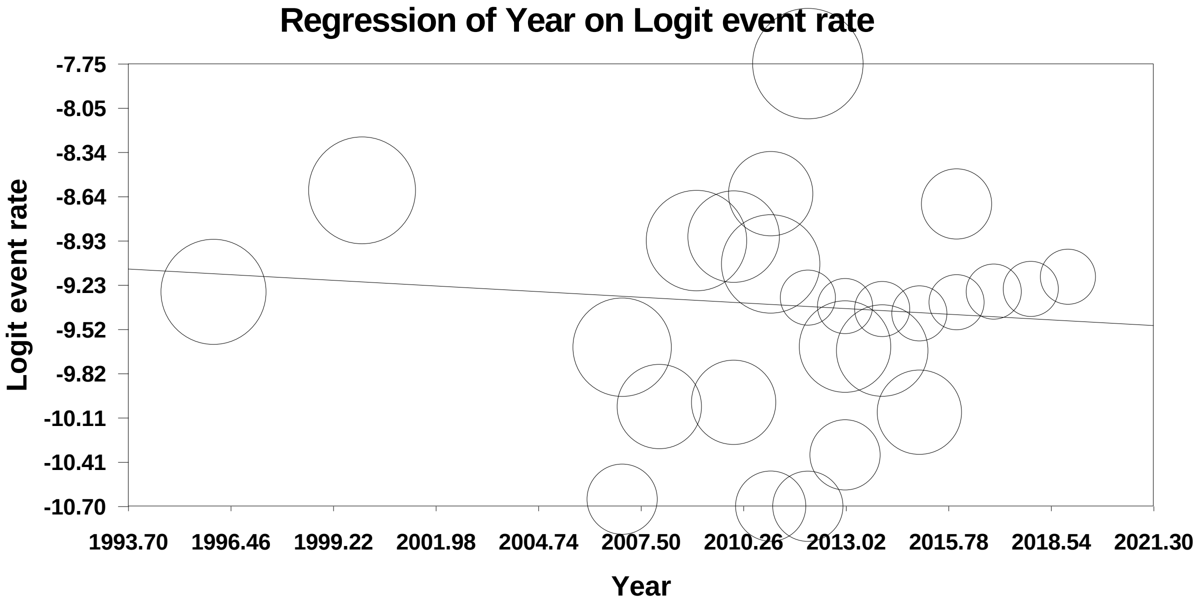

Meta-regression coefficient: -0.013, 95% CI -0.091 to 0.064, P=0.986

Fig. 6 Meta-regression model for prevalence of Screen-positive cases (a), Confirmed PKU (b), Classical PKU (c), and Hyperphenylalaninemia (HPA) (d) based on the year of study.
