# Supplementary figures and images for: The prevalence of phenylketonuria (PKU) and hyperphenylalaninemia (HPA) in Iran: a systematic review and meta-analysis
Source: Orphanet J Rare Dis. 2026 Feb 25;21:146. doi: 10.1186/s13023-026-04255-z (PMC13067558; doi:10.1186/s13023-026-04255-z)

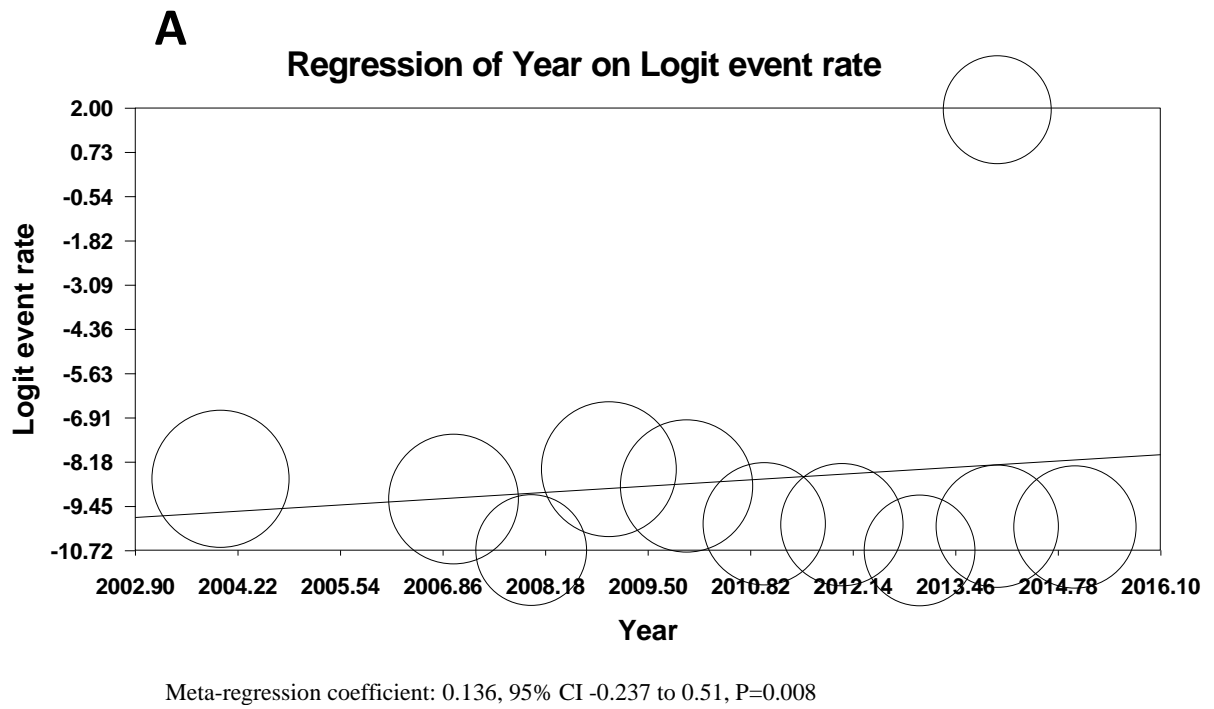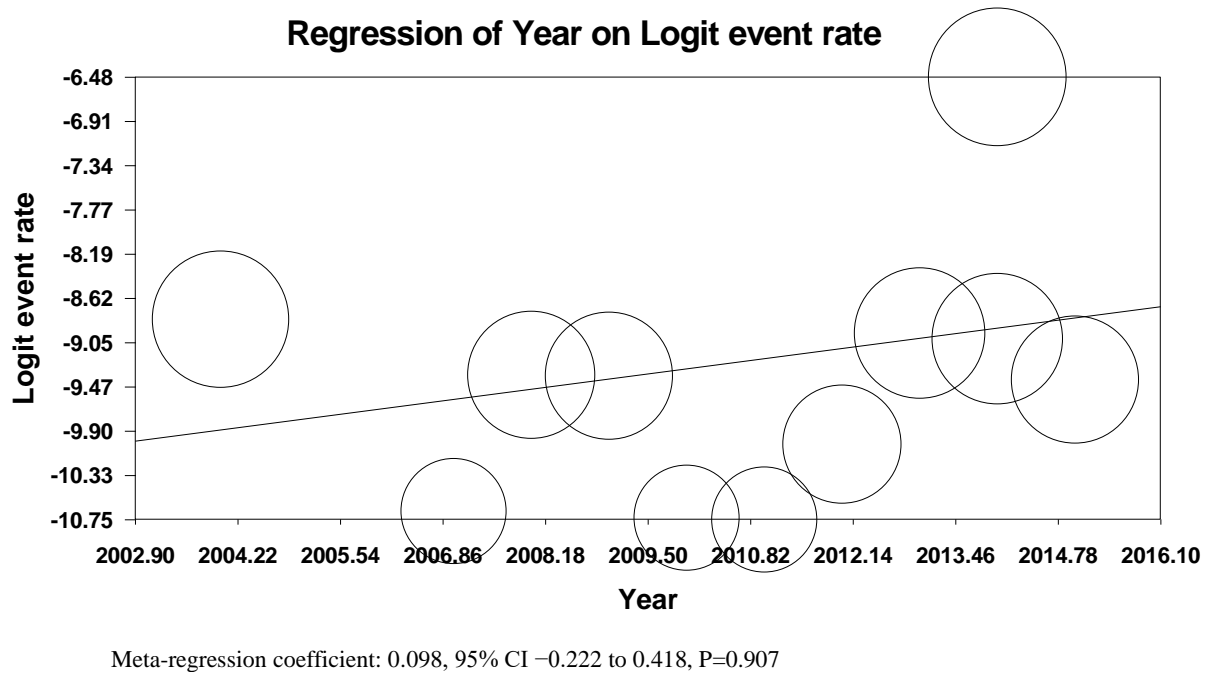

Fig. 7 Meta-regression model for prevalence of PKU in girls (a) and PKU in boys (b) based on year of study.

Supplement: Supplementary file 2 — Supplementary Material 2: Additional File 2: Fig. 7 Meta-regression model for prevalence of PKU in girls (a) and PKU in boys (b) based on year of study [file 13023_2026_4255_MOESM2_ESM.pdf]
