# Supplementary material for: The prevalence of phenylketonuria (PKU) and hyperphenylalaninemia (HPA) in Iran: a systematic review and meta-analysis
Source: Orphanet J Rare Dis. 2026 Feb 25;21:146. doi: 10.1186/s13023-026-04255-z (PMC13067558; doi:10.1186/s13023-026-04255-z)
Supplement: Supplementary file 3 — Supplementary Material 3: Additional File 3: Fig. 8 Sensitivity analysis of the prevalence of screen-positive cases in neonatal screening programs in Iran [file 13023_2026_4255_MOESM3_ESM.pdf]

Study name	Statistics with study removed					Event rate (95% CI) with study removed
	Point	Lower limit	Upper limit	Z-Value	p-Value	
Rezabeigi, D. 2023 (1)	0.000743	0.000469	0.001179037	-30.589	0.000000	
Rezabeigi, D. 2023 (2)	0.000742	0.000467	0.001176492	-30.569	0.000000	
Rezabeigi, D. 2023 (3)	0.000772	0.000488	0.001218880	-30.683	0.000000	
Rezabeigi, D. 2023 (4)	0.000765	0.000484	0.001209438	-30.659	0.000000	
Rezabeigi, D. 2023 (5)	0.000758	0.000479	0.001199753	-30.640	0.000000	
Rezabeigi, D. 2023 (6)	0.000769	0.000487	0.001215423	-30.686	0.000000	
Rezabeigi, D. 2023 (7)	0.000755	0.000477	0.001195478	-30.644	0.000000	
Rezabeigi, D. 2023 (8)	0.000748	0.000472	0.001185523	-30.630	0.000000	
Habib, A. 2010	0.000797	0.000516	0.001229861	-32.192	0.000000	
Karamifar, H. 2010	0.000802	0.000512	0.001256988	-31.097	0.000000	
Abbaskhanian, A. 2017 (1)	0.000708	0.000468	0.001071296	-34.305	0.000000	
Abbaskhanian, A. 2017 (2)	0.000738	0.000456	0.001193368	-29.354	0.000000	
Abbaskhanian, A. 2017 (3)	0.000729	0.000446	0.001190796	-28.802	0.000000	
Abbaskhanian, A. 2017 (4)	0.000770	0.000487	0.001216613	-30.678	0.000000	
Abbaskhanian, A. 2017 (5)	0.000783	0.000497	0.001233771	-30.831	0.000000	
Abbaskhanian, A. 2017 (6)	0.000794	0.000504	0.001248583	-30.846	0.000000	
Abbaskhanian, A. 2017 (7)	0.000801	0.000509	0.001260079	-30.831	0.000000	
Abbaskhanian, A. 2017 (8)	0.000798	0.000508	0.001255426	-30.855	0.000000	
Abbaskhanian, A. 2017 (9)	0.000802	0.000510	0.001261063	-30.839	0.000000	
Morovatdar, N. 2015	0.000743	0.000459	0.001203607	-29.271	0.000000	
Saadatpour, Y. 2016 (1)	0.000774	0.000490	0.001222813	-30.705	0.000000	
Saadatpour, Y. 2016 (2)	0.000805	0.000511	0.001266742	-30.750	0.000000	
Mahmoodi, M. 2015	0.000770	0.000488	0.001215045	-30.771	0.000000	
Saadatinasab, Z. 2015 (2)	0.000711	0.000444	0.001138685	-30.171	0.000000	
Saadatinasab, Z. 2015 (3)	0.000716	0.000450	0.001139376	-30.518	0.000000	
Saadatinasab, Z. 2015 (4)	0.000701	0.000443	0.001108975	-30.986	0.000000	
Saadatinasab, Z. 2015 (5)	0.000723	0.000457	0.001146174	-30.766	0.000000	
Saadatinasab, Z. 2015 (6)	0.000706	0.000446	0.001117828	-30.918	0.000000	
Saadatinasab, Z. 2015 (7)	0.000769	0.000488	0.001212448	-30.845	0.000000	
Saadatinasab, Z. 2015 (8)	0.000762	0.000482	0.001202380	-30.782	0.000000	
Saadatinasab, Z. 2015 (9)	0.000764	0.000484	0.001206570	-30.773	0.000000	
Saadatinasab, Z. 2015 (10)	0.000751	0.000475	0.001185314	-30.828	0.000000	
Saadatinasab, Z. 2015 (11)	0.000723	0.000458	0.001140828	-31.006	0.000000	
Saadatinasab, Z. 2015 (12)	0.000714	0.000452	0.001127425	-31.077	0.000000	
Badiee, M. 2014	0.000739	0.000465	0.001176689	-30.389	0.000000	
Soori, M. 2018	0.000780	0.000495	0.001229350	-30.814	0.000000	
	0.000756	0.000481	0.001187254	-31.145	0.000000	

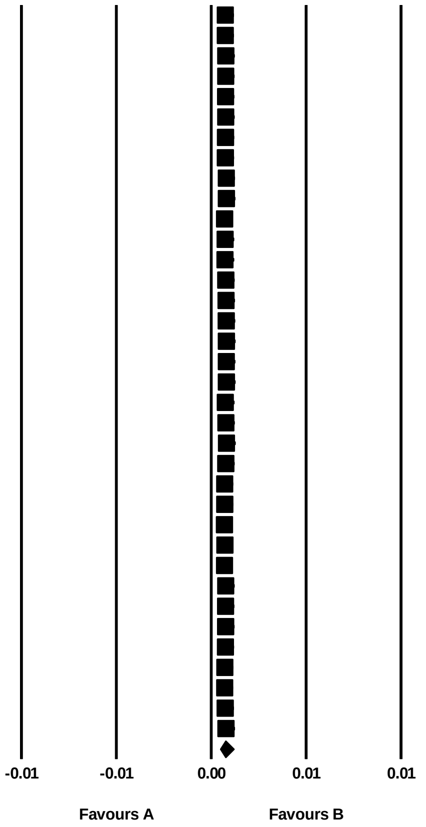

Fig. 8 Sensitivity analysis of the prevalence of screen-positive cases in neonatal screening programs in Iran.
