# Supplementary material for: The prevalence of phenylketonuria (PKU) and hyperphenylalaninemia (HPA) in Iran: a systematic review and meta-analysis
Source: Orphanet J Rare Dis. 2026 Feb 25;21:146. doi: 10.1186/s13023-026-04255-z (PMC13067558; doi:10.1186/s13023-026-04255-z)
Supplement: Supplementary file 4 — Supplementary Material 4: Additional File 4: Fig. 9 Sensitivity analysis of the prevalence of confirmed PKU in neonatal screening programs in Iran [file 13023_2026_4255_MOESM4_ESM.pdf]

Study name	Statistics for each study					Event rate and 95% CI
	Event rate	Lower limit	Upper limit	Z-Value	p-Value	
Motamedi, N. 2017 (1)	0.000120	0.000045	0.000320	-18.05	0.000000	
Motamedi, N. 2017 (2)	0.000148	0.000061	0.000354	-19.72	0.000000	
Motamedi, N. 2017 (3)	0.000206	0.000098	0.000431	-22.46	0.000000	
Motamedi, N. 2017 (4)	0.000139	0.000058	0.000334	-19.86	0.000000	
Motamedi, N. 2017 (5)	0.000171	0.000077	0.000381	-21.24	0.000000	
Motamedi, N. 2017 (6)	0.000279	0.000150	0.000519	-25.87	0.000000	
Motamedi, N. 2017 (7)	0.000161	0.000073	0.000359	-21.38	0.000000	
Motamedi, N. 2017 (8)	0.000105	0.000040	0.000281	-18.31	0.000000	
Motamedi, N. 2017 (9)	0.000381	0.000230	0.000632	-30.49	0.000000	
Motamedi, N. 2017 (10)	0.000130	0.000054	0.000311	-20.01	0.000000	
Motamedi, N. 2017 (11)	0.000191	0.000091	0.000401	-22.65	0.000000	
Rezabeigi, D. 2023 (1)	0.000090	0.000006	0.001443	-6.58	0.000000	
Rezabeigi, D. 2023 (2)	0.000513	0.000165	0.001588	-13.12	0.000000	
Rezabeigi, D. 2023 (3)	0.000084	0.000005	0.001337	-6.64	0.000000	
Rezabeigi, D. 2023 (4)	0.000163	0.000023	0.001154	-8.72	0.000000	
Rezabeigi, D. 2023 (5)	0.000168	0.000024	0.001195	-8.69	0.000000	
Rezabeigi, D. 2023 (6)	0.000094	0.000006	0.001502	-6.56	0.000000	
Rezabeigi, D. 2023 (7)	0.000192	0.000027	0.001359	-8.56	0.000000	
Rezabeigi, D. 2023 (8)	0.000208	0.000029	0.001472	-8.48	0.000000	
Habib, A. 2010	0.000160	0.000110	0.000231	-46.25	0.000000	
Senemar, S. 2009	0.000213	0.000128	0.000353	-32.74	0.000000	
RezabighiDavarani, F. 2023 (1)	0.000168	0.000063	0.000447	-17.38	0.000000	
RezabighiDavarani, F. 2023 (2)	0.000041	0.000006	0.000289	-10.11	0.000000	
RezabighiDavarani, F. 2023 (3)	0.000116	0.000037	0.000360	-15.69	0.000000	
RezabighiDavarani, F. 2023 (4)	0.000226	0.000102	0.000503	-20.56	0.000000	
RezabighiDavarani, F. 2023 (5)	0.000264	0.000126	0.000554	-21.80	0.000000	
RezabighiDavarani, F. 2023 (6)	0.000020	0.000001	0.000312	-7.67	0.000000	
RezabighiDavarani, F. 2023 (7)	0.000085	0.000021	0.000341	-13.25	0.000000	
RezabighiDavarani, F. 2023 (8)	0.000099	0.000025	0.000397	-13.03	0.000000	
Karamifar, H. 2010	0.000104	0.000052	0.000208	-25.94	0.000000	
Abbaskhanian, A. 2017 (1)	0.000047	0.000012	0.000189	-14.09	0.000000	
Abbaskhanian, A. 2017 (2)	0.000044	0.000011	0.000175	-14.19	0.000000	
Abbaskhanian, A. 2017 (3)	0.000154	0.000073	0.000323	-23.22	0.000000	
Abbaskhanian, A. 2017 (4)	0.000068	0.000022	0.000209	-16.63	0.000000	
Abbaskhanian, A. 2017 (5)	0.000023	0.000003	0.000160	-10.70	0.000000	
Abbaskhanian, A. 2017 (6)	0.000045	0.000011	0.000180	-14.15	0.000000	
Abbaskhanian, A. 2017 (7)	0.000065	0.000021	0.000202	-16.69	0.000000	
Abbaskhanian, A. 2017 (8)	0.000085	0.000032	0.000226	-18.75	0.000000	
Abbaskhanian, A. 2017 (9)	0.000063	0.000020	0.000196	-16.75	0.000000	
Ordooei, M. 2015	0.000181	0.000068	0.000481	-17.23	0.000000	
Morovatdar, N. 2015	0.000058	0.000022	0.000154	-19.52	0.000000	
Saadatpour, Y. 2016	0.000082	0.000026	0.000253	-16.30	0.000000	
ModaresSadrani, N. 2013	0.000294	0.000171	0.000506	-29.32	0.000000	
Mahmoodi, M. 2015	0.000054	0.000020	0.000144	-19.65	0.000000	
Golbahar, J. 2002	0.000404	0.000299	0.000544	-51.23	0.000000	
Moradi, K. 2014	0.000317	0.000171	0.000590	-25.47	0.000000	
Saadatinasab, Z. 2015 (2)	0.000244	0.000079	0.000756	-14.41	0.000000	
Saadatinasab, Z. 2015 (3)	0.000184	0.000026	0.001305	-8.60	0.000000	
Saadatinasab, Z. 2015 (4)	0.000903	0.000226	0.003603	-9.91	0.000000	
Saadatinasab, Z. 2015 (5)	0.000322	0.000020	0.005128	-5.68	0.000000	
Saadatinasab, Z. 2015 (6)	0.000414	0.000026	0.006586	-5.51	0.000000	
Saadatinasab, Z. 2015 (7)	0.000229	0.000014	0.003654	-5.93	0.000000	
Saadatinasab, Z. 2015 (8)	0.000258	0.000016	0.004110	-5.84	0.000000	
Saadatinasab, Z. 2015 (9)	0.000217	0.000014	0.003461	-5.96	0.000000	
Saadatinasab, Z. 2015 (10)	0.000521	0.000033	0.008277	-5.34	0.000000	
Saadatinasab, Z. 2015 (11)	0.003289	0.000205	0.050338	-4.03	0.000055	
Saadatinasab, Z. 2015 (12)	0.005747	0.000359	0.085217	-3.63	0.000280	
Badiee, M. 2014	0.000270	0.000087	0.000838	-14.23	0.000000	
Ajami, A. 2014	0.000584	0.000436	0.000783	-49.92	0.000000	
Ganji, F. 2018 (1)	0.000077	0.000011	0.000545	-9.47	0.000000	
Ganji, F. 2018 (2)	0.000204	0.000077	0.000543	-16.99	0.000000	
Ganji, F. 2018 (3)	0.000152	0.000049	0.000471	-15.23	0.000000	
Ganji, F. 2018 (4)	0.000144	0.000046	0.000445	-15.32	0.000000	
Behineh, M. 2013	0.000313	0.000078	0.001249	-11.41	0.000000	
Soori, M. 2018	0.000088	0.000005	0.001400	-6.61	0.000000	
	0.000167	0.000136	0.000205	-83.64	0.000000	

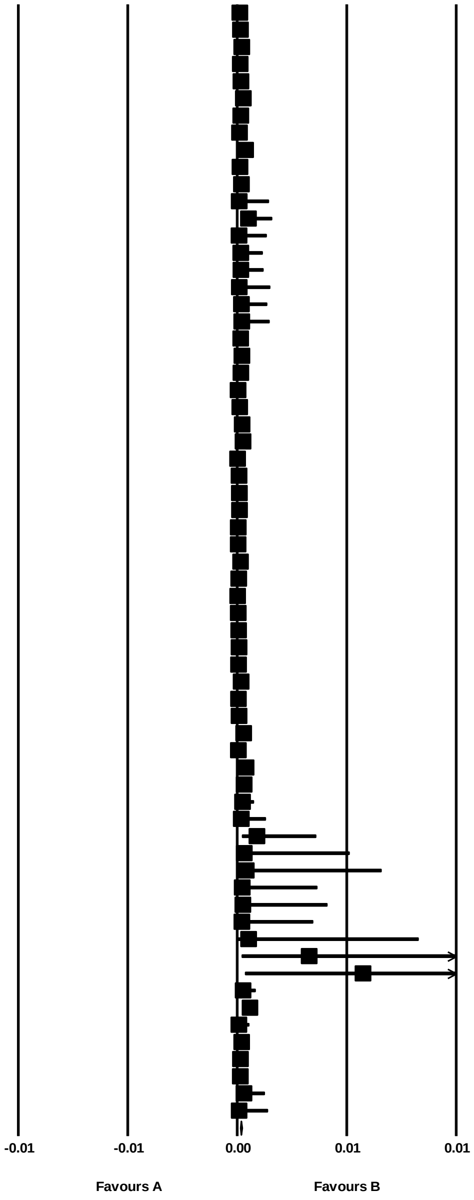

Fig. 9 Sensitivity analysis of the prevalence of confirmed PKU in neonatal screening programs in Iran.
