# Supplementary material for: The prevalence of phenylketonuria (PKU) and hyperphenylalaninemia (HPA) in Iran: a systematic review and meta-analysis
Source: Orphanet J Rare Dis. 2026 Feb 25;21:146. doi: 10.1186/s13023-026-04255-z (PMC13067558; doi:10.1186/s13023-026-04255-z)
Supplement: Supplementary file 5 — Supplementary Material 5: Additional File 5: Fig. 10 Sensitivity analysis of the prevalence of Classic PKU (a) HPA (b) in neonatal screening programs in Iran [file 13023_2026_4255_MOESM5_ESM.pdf]

A

Study name	Statistics with study removed					Event rate (95% CI) with study removed
	Point	Lower limit	Upper limit	Z-Value	p-Value	
Rezabeigi, D. 2023 (1)	0.000079	0.000050	0.000126081	-40.077	0.000000	
Rezabeigi, D. 2023 (2)	0.000072	0.000045	0.000114863	-40.249	0.000000	
Rezabeigi, D. 2023 (3)	0.000080	0.000050	0.000126285	-40.082	0.000000	
Rezabeigi, D. 2023 (4)	0.000078	0.000049	0.000123721	-39.791	0.000000	
Rezabeigi, D. 2023 (5)	0.000080	0.000050	0.000126270	-40.081	0.000000	
Rezabeigi, D. 2023 (6)	0.000079	0.000050	0.000125975	-40.076	0.000000	
Rezabeigi, D. 2023 (7)	0.000077	0.000048	0.000123049	-39.819	0.000000	
Rezabeigi, D. 2023 (8)	0.000077	0.000048	0.000122721	-39.837	0.000000	
Senemar, S. 2009	0.000085	0.000054	0.000134444	-40.489	0.000000	
Karamifar, H. 2010	0.000084	0.000053	0.000133425	-40.214	0.000000	
Abbaskhanian, A. 2017 (1)	0.000084	0.000053	0.000132377	-40.394	0.000000	
Abbaskhanian, A. 2017 (2)	0.000084	0.000053	0.000132016	-40.708	0.000000	
Abbaskhanian, A. 2017 (3)	0.000084	0.000053	0.000132724	-40.452	0.000000	
Abbaskhanian, A. 2017 (4)	0.000084	0.000053	0.000132613	-40.433	0.000000	
Abbaskhanian, A. 2017 (5)	0.000084	0.000053	0.000131926	-40.691	0.000000	
Abbaskhanian, A. 2017 (6)	0.000084	0.000053	0.000132605	-40.432	0.000000	
Abbaskhanian, A. 2017 (7)	0.000084	0.000053	0.000132034	-40.712	0.000000	
Abbaskhanian, A. 2017 (8)	0.000084	0.000054	0.000132912	-40.485	0.000000	
Abbaskhanian, A. 2017 (9)	0.000084	0.000054	0.000132935	-40.489	0.000000	
Ordooei, M. 2015	0.000082	0.000051	0.000129315	-39.990	0.000000	
Saadatpour, Y. 2016	0.000081	0.000051	0.000129688	-39.716	0.000000	
ModaresSadrani, N. 2013	0.000074	0.000045	0.000120161	-38.060	0.000000	
Golbahar, J. 2002	0.000073	0.000047	0.000114942	-41.311	0.000000	
Moradi, K. 2014	0.000072	0.000044	0.000116300	-38.651	0.000000	
Badiee, M. 2014	0.000079	0.000050	0.000126227	-39.789	0.000000	
Ajami, A. 2014	0.000073	0.000045	0.000121117	-37.318	0.000000	
Soori, M. 2018	0.000080	0.000050	0.000126163	-40.079	0.000000	
	0.000080	0.000051	0.000125944	-40.753	0.000000	

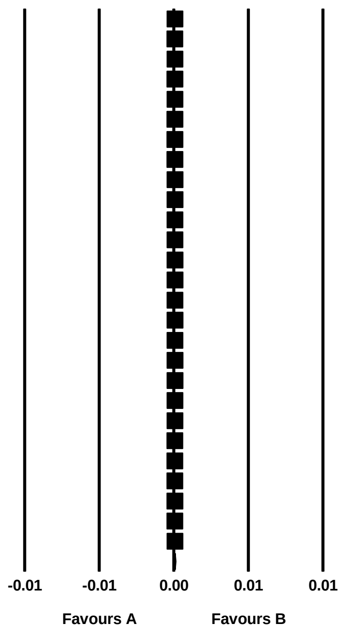

B

Study name	Statistics with study removed					Event rate (95% CI) with study removed
	Point	Lower limit	Upper limit	Z-Value	p-Value	
Rezabeigi, D. 2023 (1)	0.000088	0.000058	0.000134712	-43.526	0.000000	
Rezabeigi, D. 2023 (2)	0.000089	0.000058	0.000134844	-43.531	0.000000	
Rezabeigi, D. 2023 (3)	0.000089	0.000058	0.000134892	-43.532	0.000000	
Rezabeigi, D. 2023 (4)	0.000089	0.000058	0.000134960	-43.535	0.000000	
Rezabeigi, D. 2023 (5)	0.000087	0.000057	0.000132564	-43.235	0.000000	
Rezabeigi, D. 2023 (6)	0.000088	0.000058	0.000134617	-43.524	0.000000	
Rezabeigi, D. 2023 (7)	0.000088	0.000058	0.000134574	-43.523	0.000000	
Rezabeigi, D. 2023 (8)	0.000088	0.000058	0.000134383	-43.520	0.000000	
Senemar, S. 2009	0.000083	0.000053	0.000130009	-40.654	0.000000	
Karamifar, H. 2010	0.000091	0.000059	0.000138591	-42.832	0.000000	
Abbaskhanian, A. 2017 (1)	0.000093	0.000061	0.000140146	-43.985	0.000000	
Abbaskhanian, A. 2017 (2)	0.000092	0.000060	0.000139593	-43.451	0.000000	
Abbaskhanian, A. 2017 (3)	0.000086	0.000055	0.000132888	-41.753	0.000000	
Abbaskhanian, A. 2017 (4)	0.000092	0.000060	0.000139429	-43.414	0.000000	
Abbaskhanian, A. 2017 (5)	0.000093	0.000061	0.000140318	-44.032	0.000000	
Abbaskhanian, A. 2017 (6)	0.000093	0.000061	0.000140319	-44.032	0.000000	
Abbaskhanian, A. 2017 (7)	0.000090	0.000059	0.000137892	-42.902	0.000000	
Abbaskhanian, A. 2017 (8)	0.000090	0.000059	0.000138073	-42.936	0.000000	
Abbaskhanian, A. 2017 (9)	0.000092	0.000061	0.000139807	-43.500	0.000000	
Ordooei, M. 2015	0.000086	0.000056	0.000132848	-42.449	0.000000	
ModaresSadrani, N. 2013	0.000087	0.000056	0.000134148	-42.025	0.000000	
Golbahar, J. 2002	0.000088	0.000056	0.000135951	-41.623	0.000000	
Moradi, K. 2014	0.000092	0.000060	0.000139047	-43.724	0.000000	
Badiee, M. 2014	0.000086	0.000056	0.000131373	-42.826	0.000000	
Ajami, A. 2014	0.000095	0.000074	0.000120748	-74.691	0.000000	
Soori, M. 2018	0.000089	0.000058	0.000134784	-43.529	0.000000	
	0.000089	0.000059	0.000134166	-44.233	0.000000	

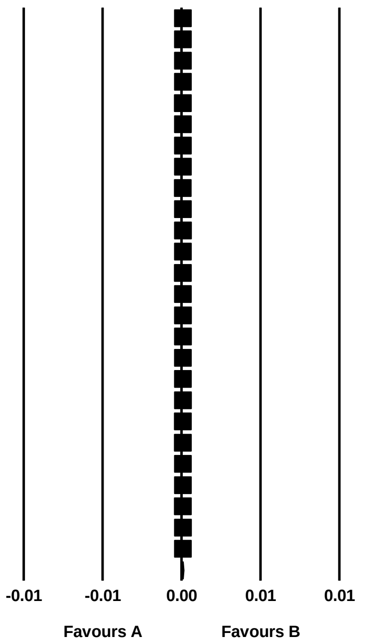

Fig. 10 Sensitivity analysis of the prevalence of Classic PKU (a) Hyperphenylalaninemia (HPA) (b) in neonatal screening programs in Iran.
