# Supplementary material for: The prevalence of phenylketonuria (PKU) and hyperphenylalaninemia (HPA) in Iran: a systematic review and meta-analysis
Source: Orphanet J Rare Dis. 2026 Feb 25;21:146. doi: 10.1186/s13023-026-04255-z (PMC13067558; doi:10.1186/s13023-026-04255-z)
Supplement: Supplementary file 6 — Supplementary Material 6: Additional File 6: Fig. 11 Sensitivity analysis of the prevalence of confirmed PKU in girls (a) and boys (b) in neonatal screening programs in Iran [file 13023_2026_4255_MOESM6_ESM.pdf]

A

Study name	Statistics with study removed				
	Point	Lower limit	Upper limit	Z-Value	p-Value
Habib, A. 2010	0.000158	0.000039	0.000645	-12.183	0.000000
Abbaskhanian, A. 2017 (1)	0.000162	0.000050	0.000531	-14.422	0.000000
Abbaskhanian, A. 2017 (2)	0.000175	0.000058	0.000532	-15.250	0.000000
Abbaskhanian, A. 2017 (3)	0.000149	0.000042	0.000535	-13.506	0.000000
Abbaskhanian, A. 2017 (4)	0.000157	0.000046	0.000531	-14.047	0.000000
Abbaskhanian, A. 2017 (5)	0.000171	0.000055	0.000535	-14.900	0.000000
Abbaskhanian, A. 2017 (6)	0.000171	0.000055	0.000536	-14.904	0.000000
Abbaskhanian, A. 2017 (7)	0.000175	0.000058	0.000532	-15.251	0.000000
Abbaskhanian, A. 2017 (8)	0.000172	0.000055	0.000538	-14.909	0.000000
Abbaskhanian, A. 2017 (9)	0.000172	0.000055	0.000538	-14.912	0.000000
Saadatpour, Y. 2016	0.000118	0.000076	0.000183	-40.149	0.000000
	0.000152	0.000052	0.000442	-16.128	0.000000

Event rate (95% CI) with study removed

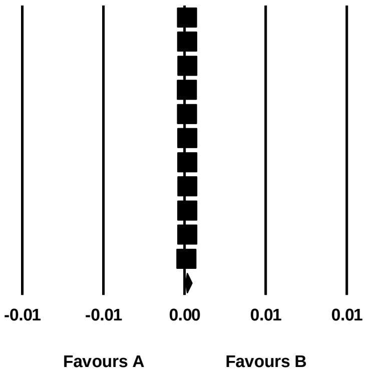

B

Study name	Statistics with study removed				
	Point	Lower limit	Upper limit	Z-Value	p-Value
Habib, A. 2010	0.000090	0.000024	0.000331	-14.022	0.000000
Abbaskhanian, A. 2017 (1)	0.000109	0.000035	0.000341	-15.647	0.000000
Abbaskhanian, A. 2017 (2)	0.000099	0.000031	0.000321	-15.353	0.000000
Abbaskhanian, A. 2017 (3)	0.000099	0.000031	0.000321	-15.356	0.000000
Abbaskhanian, A. 2017 (4)	0.000109	0.000035	0.000342	-15.651	0.000000
Abbaskhanian, A. 2017 (5)	0.000109	0.000035	0.000342	-15.652	0.000000
Abbaskhanian, A. 2017 (6)	0.000105	0.000033	0.000335	-15.529	0.000000
Abbaskhanian, A. 2017 (7)	0.000094	0.000028	0.000312	-15.151	0.000000
Abbaskhanian, A. 2017 (8)	0.000095	0.000029	0.000314	-15.169	0.000000
Abbaskhanian, A. 2017 (9)	0.000100	0.000031	0.000322	-15.365	0.000000
Saadatpour, Y. 2016	0.000109	0.000075	0.000158	-47.850	0.000000
	0.000098	0.000032	0.000298	-16.247	0.000000

Event rate (95% CI) with study removed

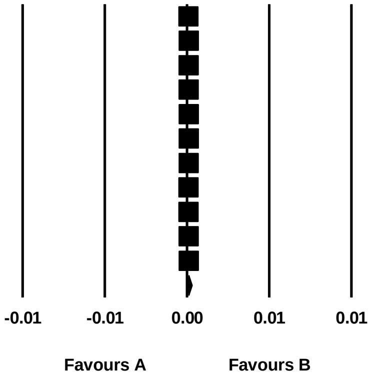

Fig. 11 Sensitivity analysis of the prevalence of confirmed PKU in girls (a) and boys (b) in neonatal screening programs in Iran.
