# Supplementary material for: The prevalence of phenylketonuria (PKU) and hyperphenylalaninemia (HPA) in Iran: a systematic review and meta-analysis
Source: Orphanet J Rare Dis. 2026 Feb 25;21:146. doi: 10.1186/s13023-026-04255-z (PMC13067558; doi:10.1186/s13023-026-04255-z)
Supplement: Supplementary file 7 — Supplementary Material 7: Additional File 7: Fig. 12 Publication bias for the prevalence of Screen-positive cases (a), Confirmed PKU (b), Classical PKU (c), and HPA (d) based on the year of study [file 13023_2026_4255_MOESM7_ESM.pdf]

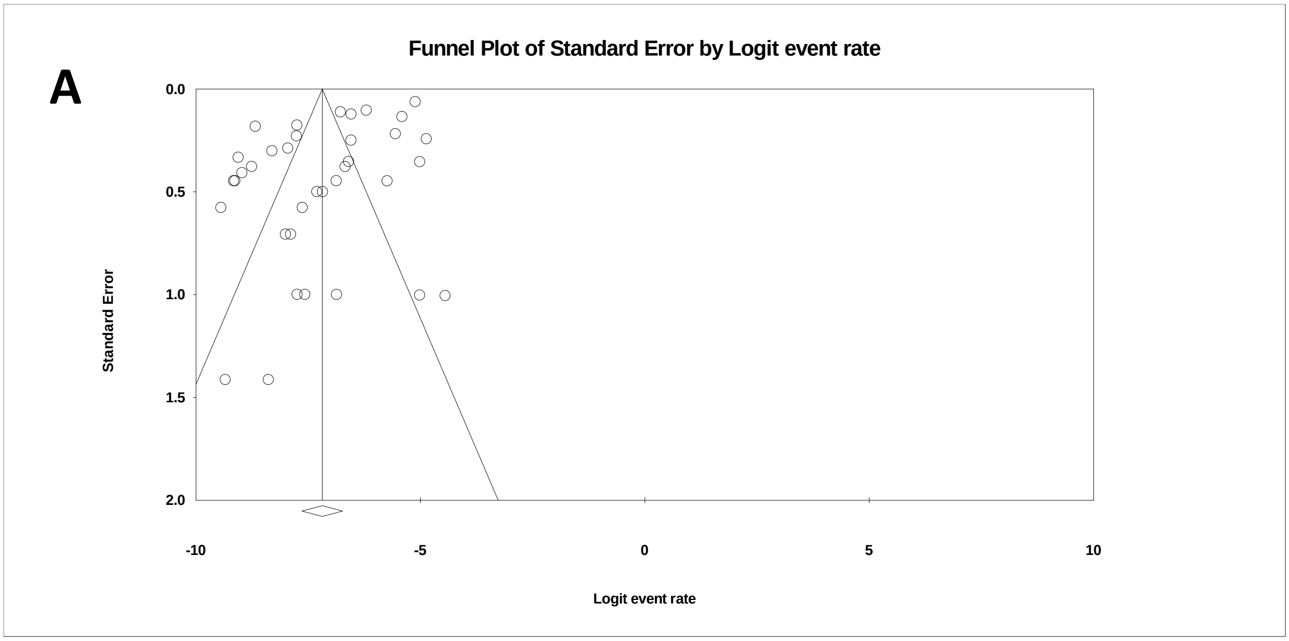

A
Funnel Plot of Standard Error by Logit event rate
Standard Error
0.0
0.5
1.0
1.5
2.0
-10
-5
0
5
10
Logit event rate

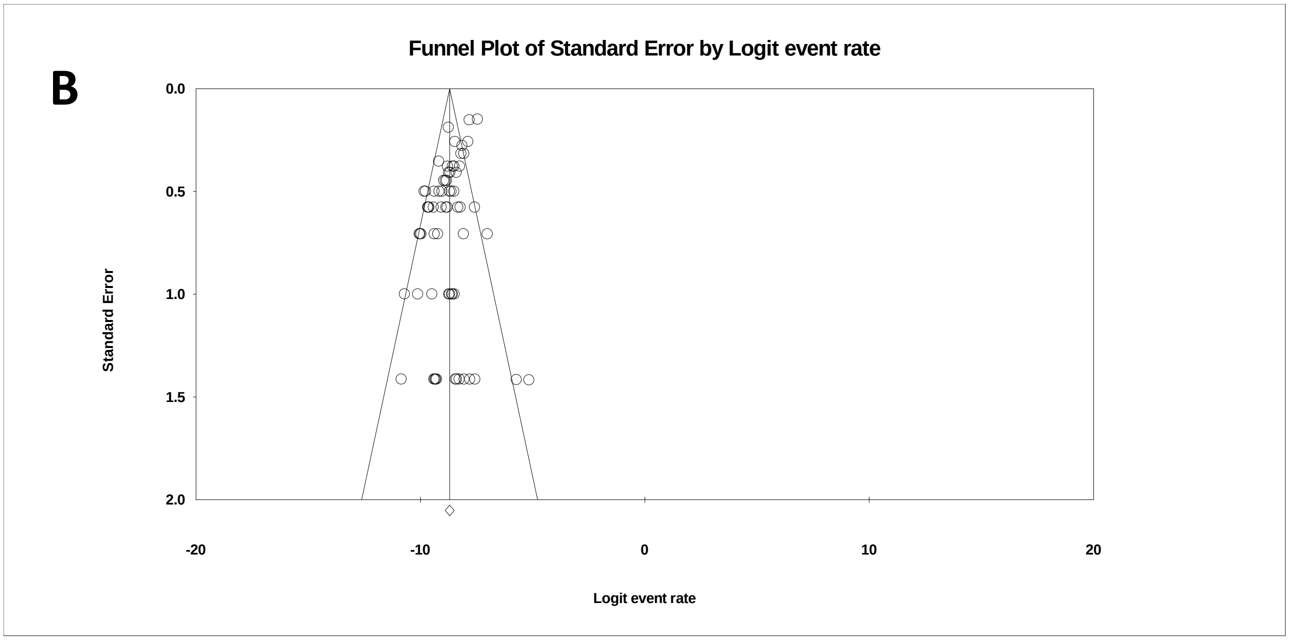

B
Funnel Plot of Standard Error by Logit event rate
Standard Error
0.0
0.5
1.0
1.5
2.0
-20
-10
0
10
20
Logit event rate

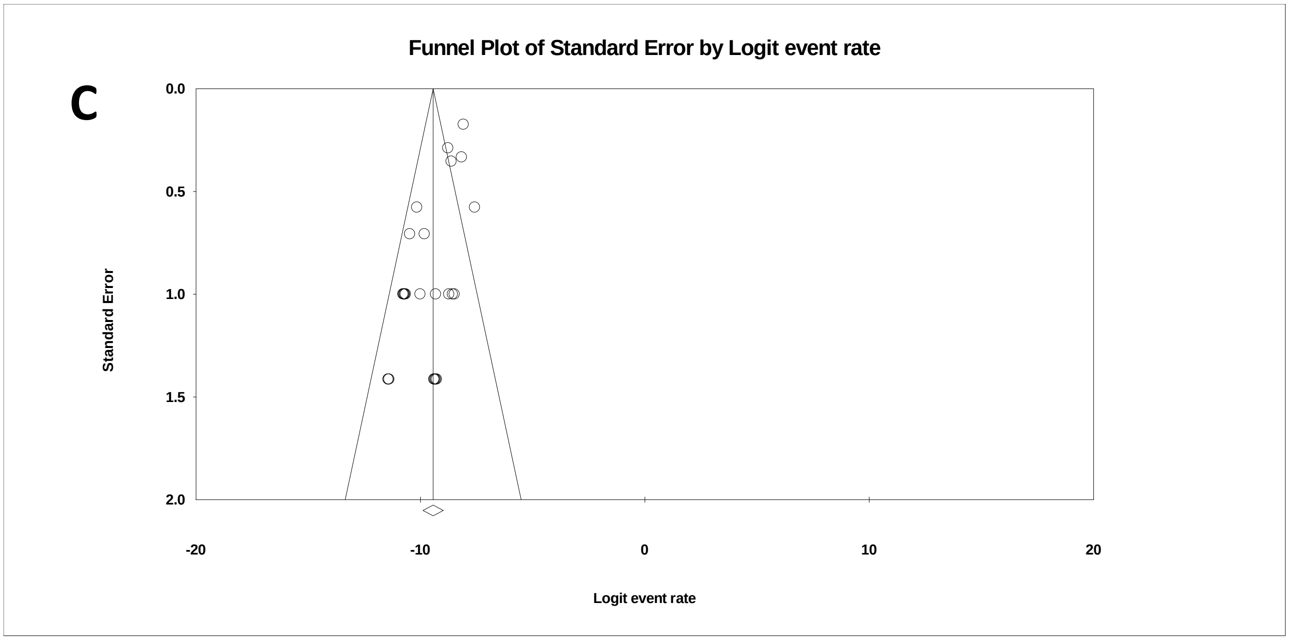

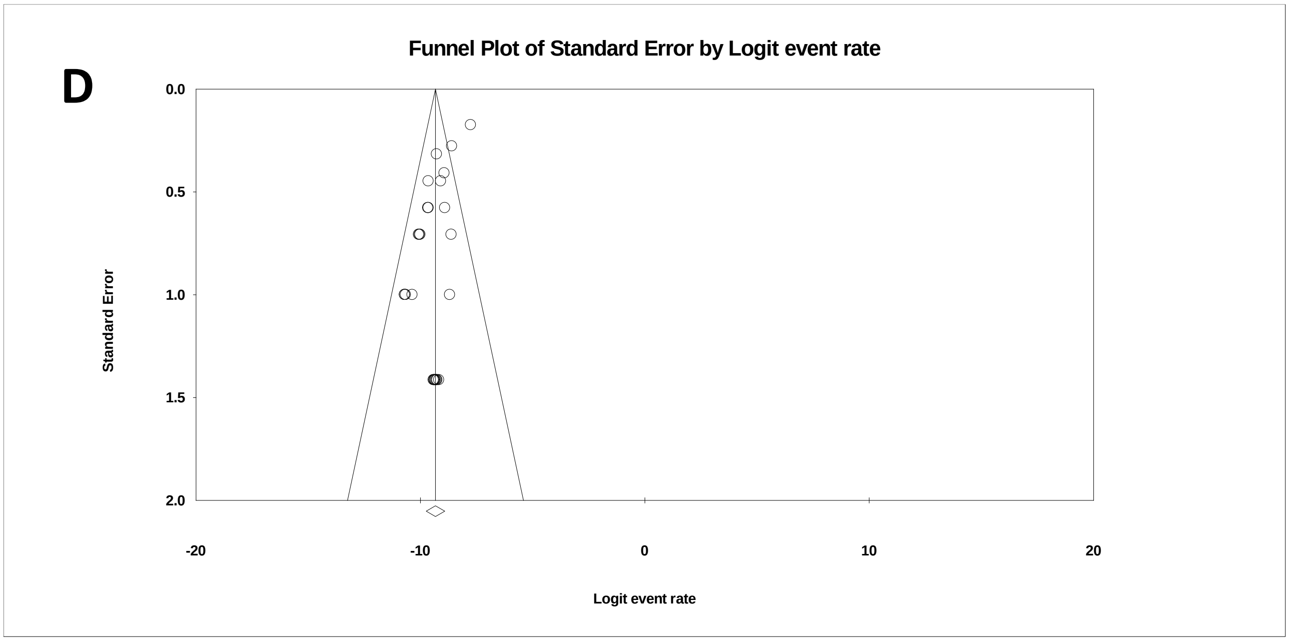

Fig. 12 Publication bias for the prevalence of Screen-positive cases (a), Confirmed PKU (b), Classical PKU (c), and Hyperphenylalaninemia (HPA) (d) based on the year of study.
