# Supplementary material for: The prevalence of phenylketonuria (PKU) and hyperphenylalaninemia (HPA) in Iran: a systematic review and meta-analysis
Source: Orphanet J Rare Dis. 2026 Feb 25;21:146. doi: 10.1186/s13023-026-04255-z (PMC13067558; doi:10.1186/s13023-026-04255-z)
Supplement: Supplementary file 8 — Supplementary Material 8: Additional File 8: Fig. 13 Publication bias for prevalence of confirmed PKU in girls (a), confirmed PKU in boys (b), and girls to boys odds ratio (c) based on year of study [file 13023_2026_4255_MOESM8_ESM.pdf]

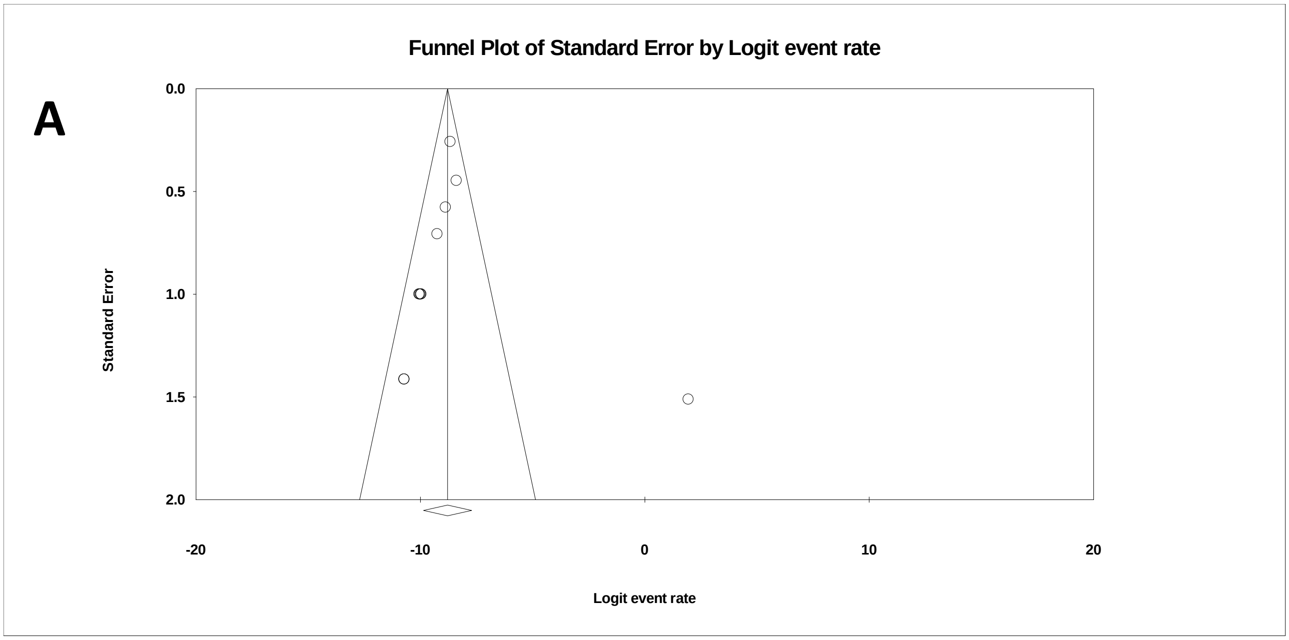
A
Funnel Plot of Standard Error by Logit event rate
0.0
0.5
1.0
1.5
2.0
Standard Error
-20
-10
0
10
20
Logit event rate

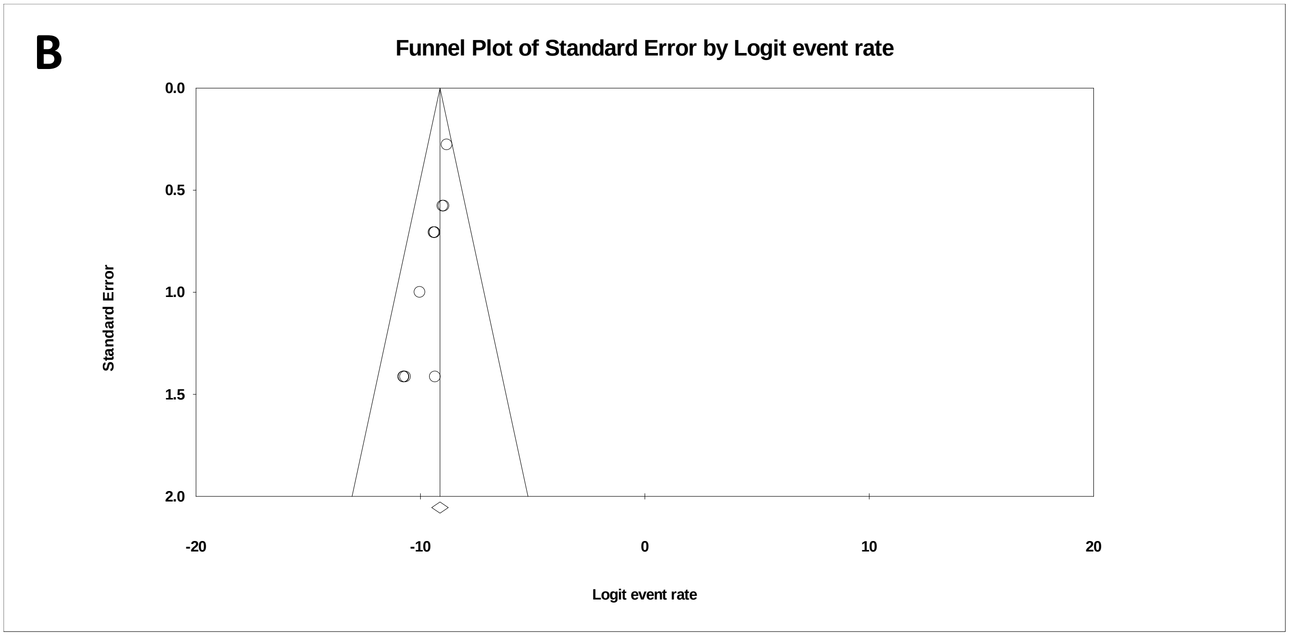
B
Funnel Plot of Standard Error by Logit event rate
0.0
0.5
1.0
1.5
2.0
Standard Error
-20
-10
0
10
20
Logit event rate

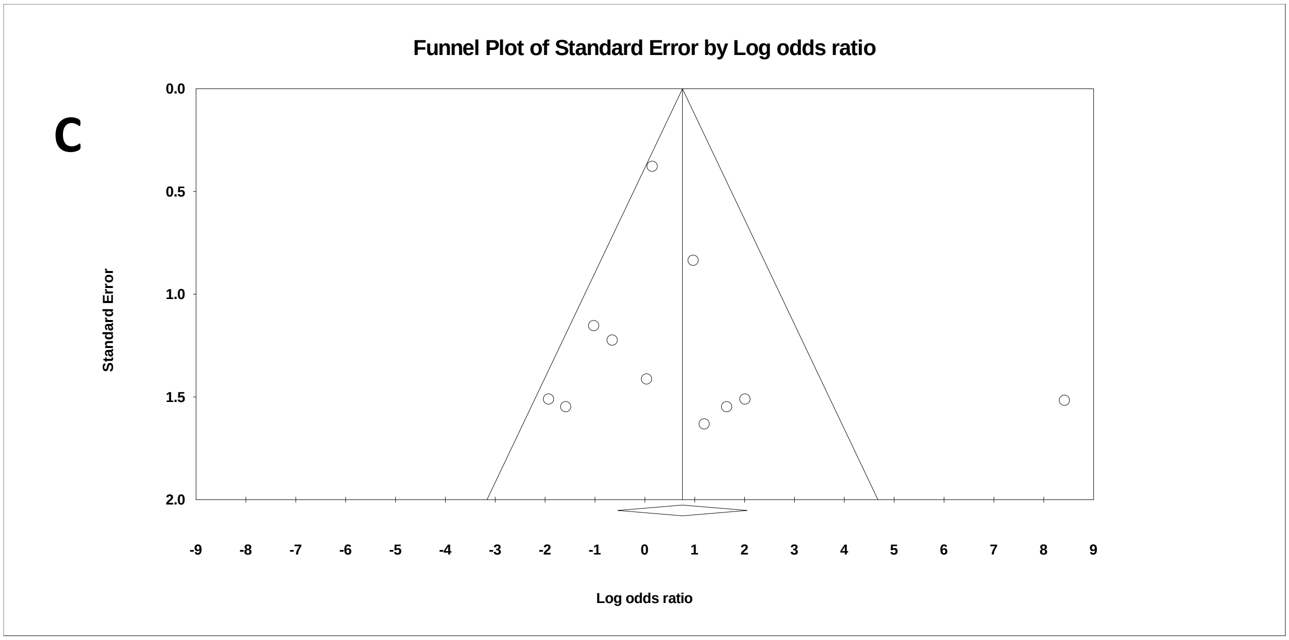

Fig. 13 Publication bias for prevalence of confirmed PKU in girls (a), confirmed PKU in boys (b), and girls to boys odds ratio (c) based on year of study.
